# Supplementary material for: Hematopoietic mosaic chromosomal alterations are pleiotropic drivers of inflammaging, multimorbidity, and mortality
Source: medRxiv. 2026 Jul 1:2026.06.24.26356446. Preprint. [Version 1] doi: 10.64898/2026.06.24.26356446 (PMC13345432; doi:10.64898/2026.06.24.26356446)

medRxiv preprint doi: https://doi.org/10.64898/2026.06.24.26356446; this version posted July 1, 2026. The copyright holder for this preprint (which was not certified by peer review) is the author/funder, who has granted medRxiv a license to display the preprint in perpetuity.
It is made available under a CC-BY 4.0 International license .

Supplementary Information

Hematopoietic mosaic chromosomal alterations are pleiotropic drivers of inflammaging, multimorbidity, and mortality

Nicole D. Vincelette[1,†], Qianxing Mo[2], Chia-Ho Cheng[2], Junyoung Park[1], Andrew T. Kuykendall[1], Ling Zhang[3], Jungwon Moon[1], Tiffany N. Razabdouski[1], Erika A. Eksioglu[4], Felyschia M. Lledo[5], Peter R. Papenhausen[3], Zhuoer Xie[1], Onyee Chan[1], David A. Sallman[1], Javier Pinilla-Ibarz[1], Daniel J. Murphy[6,7], Rami S. Komrokji[1], John L. Cleveland[8], Xiaoqing Yu[2], and Seongseok Yun[1,†]

[1]Malignant Hematology Department, H. Lee Moffitt Cancer Center and Research Institute, Tampa, FL, USA, [2]Department of Biostatistics and Bioinformatics, H. Lee Moffitt Cancer Center and Research Institute, Tampa, FL, USA. [3]Department of Pathology and Laboratory Medicine, H. Lee Moffitt Cancer Center and Research Institute, Tampa, FL, USA. [4]Department of Immunology, H. Lee Moffitt Cancer Center and Research Institute, Tampa, FL, USA, [5]NTRO Clinical Research Operations, H. Lee Moffitt Cancer Center and Research Institute, Tampa, FL, USA. [6]Cancer Research UK Scotland Institute, Glasgow, United Kingdom. [7]School of Cancer Sciences, University of Glasgow, Glasgow, United Kingdom. [8]Department of Tumor Microenvironment & Metastasis, H. Lee Moffitt Cancer Center and Research Institute, Tampa, FL, USA.

†N.D.V. and S.Y. contributed equally as co-first authors and share corresponding authorship

Correspondence to:
Nicole D. Vincelette PhD
Malignant Hematology Department
H. Lee Moffitt Cancer Center and Research Institute
12902 USF Magnolia Drive
Tampa, FL 33612
Email: Nicole.Vincelette@moffitt.org

Seongseok Yun MD PhD
Malignant Hematology Department
H. Lee Moffitt Cancer Center and Research Institute
12902 USF Magnolia Drive
Tampa, FL 33612
Email: Seongseok.Yun@moffitt.org

Supplementary Information includes:
Supplementary Figures S1-S19
Supplementary Table S1-S14

medRxiv preprint doi: https://doi.org/10.64898/2026.06.24.26356446; this version posted July 1, 2026. The copyright holder for this preprint (which was not certified by peer review) is the author/funder, who has granted medRxiv a license to display the preprint in perpetuity.
It is made available under a CC-BY 4.0 International license .

Table S1: Baseline demographic and laboratory profiles in UKBB cohort

Variables	mCA (n=42,390)	No mCA (n=410,204)	Total (n=452,594)
**Sex (%)**			
Female	14,659 (34.6)	240,220 (58.6)	254,879 (56.3)
Male	27,731 (65.4)	169,984 (41.4)	197,715 (43.7)
**Age**, median (IQR)			
At recruitment	63 (59-66)	57 (49-62)	57 (50-63)
At mCA assessment	63 (59-67)	57 (50-63)	58 (50-63)
**Follow up**, median (IQR)	14.7 (13.9-15.5)	14.9 (14.1-15.6)	14.9 (14.1-15.6)
**Lifestyle** (%)			
Smoking history	23,427 (55.3)	177,694 (43.3)	210,121 (44.4)
Alcohol use	40,889 (96.5)	390,150 (95.1)	431,039 (95.2)
**CBC**, median (IQR)			
WBC, cells/μL	6.9 (5.9-8.2)	6.6 (5.6-7.8)	6.6 (5.6-7.8)
Neutrophil, cells/μL	4.2 (3.4-5.1)	4.0 (3.2-4.9)	4.0 (3.3-5.0)
Hemoglobin, g/dL	14.5 (13.6-15.3)	14.1 (13.3-15.0)	14.1 (13.3-15.0)
Platelet, K/μL	242.5 (208.2-282.0)	249.1 (214.5-288.1)	248.7 (214.0-287.9)

Abbreviations: mCA (mosaic chromosomal alteration), IQR (interquartile range), CBC (complete blood count), WBC (white blood cells)

medRxiv preprint doi: https://doi.org/10.64898/2026.06.24.26356446; this version posted July 1, 2026. The copyright holder for this preprint (which was not certified by peer review) is the author/funder, who has granted medRxiv a license to display the preprint in perpetuity.
It is made available under a CC-BY 4.0 International license .

Table S2: Incident disease risk by ICD-10 chapter based on mCA status

Table S3: Incident disease risk by ICD-10 chapter based on individual mCA subtype

Table S4: Incident inflammaging-related disease risk by organ system based on mCA status

Table S5: Incident inflammaging-related disease risk by organ system based on individual mCA subtype

Table S6: Dose-response analysis of incident disease risk by MCF for individual mCA subtypes

Table S7: All-cause mortality risk by mCA subtype

Table S8: Cause-specific mortality risk by ICD-10 chapter based on mCA status

Table S9: Incident disease risk by ICD-10 chapter based on mCA status excluding CHIP participants

Table S10: All-cause mortality risk by mCA subtype excluding CHIP participants

Table S11: Cytoband-level disease associations identified by LASSO-Cox regression with HR estimates

Table S12: Gene annotations for disease-associated cytobands

Table S13: Differentially expressed genes in 8+ HSCs compared to healthy donor HSCs

Table S14: Differentially expressed genes in 1+ HSCs compared to healthy donor HSCs

medRxiv preprint doi: https://doi.org/10.64898/2026.06.24.26356446; this version posted July 1, 2026. The copyright holder for this preprint (which was not certified by peer review) is the author/funder, who has granted medRxiv a license to display the preprint in perpetuity.
It is made available under a CC-BY 4.0 International license .

Supplementary Fig. 1. Study design and analytical workflow. Schematic overview of the analytical pipeline. mCA calls were obtained from Loh et al.[20]; somatic mutation (CHIP) data were obtained from the Vlasschaert et al. study[45]. Left panels show the sequential stages of analysis: (i) UK Biobank cohort filtering and mCA classification, (ii) epidemiological characterization of mCA-disease associations, (iii) cytoband-level mapping to prioritize disease-associated loci and to identify candidate genes, (iv) experimental interrogation of selected candidate genes using scRNA-seq and mouse models, and (v) clinical implications. Right panels detail the statistical approaches used at each stage, including multiple testing correction, event thresholds for model stability, mCA homogeneity assessment metrics, and LASSO-penalized Cox regression parameters.

medRxiv preprint doi: https://doi.org/10.64898/2026.06.24.26356446; this version posted July 1, 2026. The copyright holder for this preprint (which was not certified by peer review) is the author/funder, who has granted medRxiv a license to display the preprint in perpetuity.
It is made available under a CC-BY 4.0 International license .

Supplementary Fig. 2. Demographic and mCA characteristics of the UK Biobank cohort. **a**, Sex distribution of the study cohort (n=452,594). **b-c**, Age distribution at recruitment (**b**) and at mCA assessment (**c**) by sex (red, female; blue, male). **d**, Distribution of mCA types among all detected events (n=45,536): loss (n=34,686; 76.2%), CNLOH (n=8,309; 18.2%), and gain (n=2,541; 5.6%). **e**, Number of mCAs per individual among mCA carriers; inset shows distribution for individuals with ≥4 mCAs. **f**, Frequency of individual mCA subtypes (log scale); colors indicate mCA type (red, gain; blue, loss; orange, CNLOH). **g-j**, Mosaic cell fraction (MCF) distribution by mCA type (**g**) and by chromosome for gain (**h**), loss (**i**), and CNLOH (**j**). Box plots show median and interquartile range.

medRxiv preprint doi: https://doi.org/10.64898/2026.06.24.26356446; this version posted July 1, 2026. The copyright holder for this preprint (which was not certified by peer review) is the author/funder, who has granted medRxiv a license to display the preprint in perpetuity.
It is made available under a CC-BY 4.0 International license .

Supplementary Fig. 3. Cross-sectional associations between mCAs and baseline demographic, lifestyle, laboratory, and inflammatory parameters. a, Associations between mCA subtypes and demographic and lifestyle factors. **b**, Associations between mCA subtypes and complete blood count parameters. **c**, Associations between mCA subtypes and serum biochemistry parameters. Heatmaps display $\log_2$-transformed odds ratios (ORs) from logistic regression; red indicates positive associations and blue indicates negative associations. For demographic and lifestyle factors, univariate logistic regression was performed. For blood count and biochemistry parameters, models were adjusted for age and sex, with values log-transformed prior to analysis. Rows represent mCA subtypes: all mCA carriers, complex mCA (≥3 chromosomes affected), small mCA (MCF <10%), large mCA (MCF ≥10%), and mCA classified by copy number change (gain, loss, or CNLOH). Multiple testing was corrected using the Benjamini-Hochberg method. **d-f,** Volcano plots showing cross-sectional differences in circulating cytokine and alarmin levels between no mCA controls (n=44,039) and mCA carriers (n=4,678) (**d**), large mCA (n=1,586) (**e**), or complex mCA (n=48) (**f**). Cytokine levels were measured using the Olink platform and expressed as Normalized Protein eXpression (NPX) values. The x-axis shows Cohen's d effect size, and the y-axis shows $-\log_{10}$(Wilcoxon p-value). Points are colored red (increased in mCA group, p<0.05), blue (decreased in mCA group, p<0.05), or gray (not significant, p≥0.05). Horizontal dashed line indicates p=0.05; vertical dashed line indicates no effect (Cohen's d=0).

medRxiv preprint doi: https://doi.org/10.64898/2026.06.24.26356446; this version posted July 1, 2026. The copyright holder for this preprint (which was not certified by peer review) is the author/funder, who has granted medRxiv a license to display the preprint in perpetuity.
It is made available under a CC-BY 4.0 International license .

Supplementary Fig. 4. Baseline disease burden and cumulative incidence of diseases by ICD-10 chapter. a, Time interval from first disease diagnosis to mCA assessment among participants with at least one baseline diagnosis, comparing mCA carriers (n=27,411) vs. non-carriers (n=250,664). Box plots show median and interquartile range (IQR); P value was calculated using the Wilcoxon rank-sum test. **b**, Distribution of baseline diseases prevalence across ICD-10 chapters in mCA carriers vs. non-carriers; P value calculated using chi-square test. **c-o**, Cumulative incidence of incident diseases by ICD-10 chapter comparing mCA carriers (red) vs. non-carriers (blue): hematologic disorders (**c**), neoplasms (**d**), respiratory (**e**), circulatory (**f**), infectious (**g**), endocrine/metabolic (**h**), mental/behavioral (**i**), nervous system (**j**), eye and ear (**k**), digestive (**l**), skin/subcutaneous (**m**), musculoskeletal (**n**), and genitourinary (**o**). Shaded areas represent 95% CIs; P values were calculated using Gray's test with death treated as a competing event.

medRxiv preprint doi: https://doi.org/10.64898/2026.06.24.26356446; this version posted July 1, 2026. The copyright holder for this preprint (which was not certified by peer review) is the author/funder, who has granted medRxiv a license to display the preprint in perpetuity.
It is made available under a CC-BY 4.0 International license .

Supplementary Fig. 5. Incident disease risk by individual mCA subtype across ICD-10 chapters. a-m, Forest plots showing HRs with 95% CIs for incident disease risk by individual mCA subtype across ICD-10 disease chapters: hematologic disorders (**a**), neoplasms (**b**), respiratory (**c**), circulatory (**d**), infectious (**e**), endocrine/metabolic (**f**), mental/behavioral (**g**), nervous system (**h**), eye and ear (**i**), digestive (**j**), skin/subcutaneous (**k**), musculoskeletal (**l**), and genitourinary (**m**). Colors indicate CNV type (red, gain; blue, loss; gray, CNLOH). Fine-Gray competing risk regression was used with death as a competing event, adjusted for age, sex, and baseline disease burden. Analyses were restricted to mCA-disease combinations with ≥30 total participants, ≥5 events per group. Multiple testing was corrected using the Benjamini-Hochberg method.

medRxiv preprint doi: https://doi.org/10.64898/2026.06.24.26356446; this version posted July 1, 2026. The copyright holder for this preprint (which was not certified by peer review) is the author/funder, who has granted medRxiv a license to display the preprint in perpetuity.
It is made available under a CC-BY 4.0 International license .

Supplementary Fig. 6. Incident inflammaging-related disease risk by individual mCA subtype. a-l, Heatmaps showing $\log_2$-transformed HRs for incident inflammaging-related diseases by mCA subtype across organ system categories: musculoskeletal (**a**), endocrine and metabolic (**b**), cardiovascular (**c**), respiratory (**d**), neoplasms (**e**), hematologic (**f**), gastrointestinal and hepatobiliary (**g**), renal (**h**), vision and hearing (**i**), skin and subcutaneous (**j**), neurologic (**k**), and all categories combined (**l**). Red indicates increased risk (HR>1) and blue indicates decreased risk (HR<1). The "Combined" row at the bottom of each panel represents the category-level combined endpoint. Hierarchical clustering was applied to columns; the Combined row was excluded from row clustering. Fine-Gray competing risk regression was used with death as a competing event, adjusted for age, sex, and baseline disease burden. Analyses were restricted to mCA-disease combinations with ≥30 total events and ≥5 events per group. Multiple testing was corrected using the Benjamini-Hochberg method.

medRxiv preprint doi: https://doi.org/10.64898/2026.06.24.26356446; this version posted July 1, 2026. The copyright holder for this preprint (which was not certified by peer review) is the author/funder, who has granted medRxiv a license to display the preprint in perpetuity.
It is made available under a CC-BY 4.0 International license .

Supplementary Fig. 7. Additional dose-response relationships and mediation analyses. a-l, Dose-response relationships between MCF and incident disease risk for selected mCA-disease pairs using 5% binning (odd panels) and natural cubic spline regression (df=3) (even panels): 13- and hematologic disorders (**a-b**), Y- and hematologic disorders (**c-d**), 12+ and respiratory disorders (**e-f**), 13- and neoplasms (**g-h**), Y- and respiratory disorders (**i-j**), and Y- and mental/behavioral disorders (**k-l**). In binning plots, dot size reflects sample size per bin; in spline plots, shaded areas represent 95% CIs. Dashed horizontal lines indicate HR=1. **m-p**, Mediation analysis of 13- on actinic keratosis risk via CLL. CLL-free survival comparing 13- carriers vs. non-carriers (**m**) and actinic keratosis-free survival comparing individuals with vs. without CLL (**n**); shaded areas represent 95% CIs and P values were calculated using the log-rank test. Adjusted absolute risk increase showing average causal mediation effect (ACME), average direct effect (ADE), and total effect with 95% CIs (**o**). Path diagram summarizing statistically inferred direct and mediated effects; dashed arrow indicates non-significant direct effect (**p**). **q-t**, Mediation analysis of 12+ on chronic bronchitis risk via CLL, displayed as in m-p; solid arrows indicate significant associations. All models were adjusted for age, sex, and baseline disease burden.

medRxiv preprint doi: https://doi.org/10.64898/2026.06.24.26356446; this version posted July 1, 2026. The copyright holder for this preprint (which was not certified by peer review) is the author/funder, who has granted medRxiv a license to display the preprint in perpetuity.
It is made available under a CC-BY 4.0 International license .

Supplementary Fig. 8. Cause-specific mortality by ICD-10 chapter. a-k, Cumulative incidence of death by ICD-10 chapter comparing mCA carriers (red) vs. non-carriers (blue): respiratory (**a**), circulatory (**b**), infectious (**c**), endocrine/metabolic (**d**), mental/behavioral (**e**), nervous system (**f**), eye and ear (**g**), digestive (**h**), skin/subcutaneous (**i**), musculoskeletal (**j**), and genitourinary (**k**). Shaded areas represent 95% CIs; P values were calculated using Gray's test.

medRxiv preprint doi: https://doi.org/10.64898/2026.06.24.26356446; this version posted July 1, 2026. The copyright holder for this preprint (which was not certified by peer review) is the author/funder, who has granted medRxiv a license to display the preprint in perpetuity.
It is made available under a CC-BY 4.0 International license .

Supplementary Fig. 9. Classification of homogeneous vs. heterogeneous mCAs. a-d, Four quantitative metrics used to classify mCA subtypes as homogeneous or heterogeneous: homogeneity index (cross-sample consistency) (**a**), Gini coefficient (coverage distribution) (**b**), normalized coverage entropy (signal dispersion) (**c**), and mean coverage (bin-level representation) (**d**). Red dashed lines indicate pre-defined classification thresholds: homogeneity index >0.9, Gini coefficient <0.25, normalized entropy <0.4, and mean coverage >0.75. All thresholds were pre-specified prior to analysis. mCAs meeting all four criteria were classified as homogeneous (red bars: 12+, 14+, 15+, 18+, X−); all others were classified as heterogeneous (blue bars). Error bars represent 95% CIs derived from bootstrap resampling (1,000 iterations). Homogeneous mCAs represent whole-chromosome or whole-arm events with relatively uniform genomic boundaries across carriers, while heterogeneous mCAs exhibit variable breakpoints amenable to cytoband-level resolution.

medRxiv preprint doi: https://doi.org/10.64898/2026.06.24.26356446; this version posted July 1, 2026. The copyright holder for this preprint (which was not certified by peer review) is the author/funder, who has granted medRxiv a license to display the preprint in perpetuity.
It is made available under a CC-BY 4.0 International license .

Supplementary Fig. 10. Validation of LASSO-penalized Cox regression via bootstrap stability analysis. **a**, Circos plot displaying cytobands with ≥70% bootstrap selection frequency across disease categories. Point size reflects LASSO coefficient magnitude; shape indicates CNV type; color denotes disease category. **b**, Heatmap circos plot showing the same cytobands, with concentric rings representing disease categories, color indicating coefficient direction (red, increased risk; blue, reduced risk), and opacity reflecting bootstrap frequency. **c**, Scatter plot of LASSO coefficients vs. Cox log(HR), demonstrating positive correlation (r=0.561) and 94% direction concordance. Points are colored by bootstrap stability category (red, ≥70%; pink, 50-70%; gray, <50%). **d**, Bootstrap selection frequency vs. Cox FDR significance (-log10 q-value), illustrating weak correlation (r=0.126) as FDR-significant associations span the full range of bootstrap stability. Features with low bootstrap stability but high FDR significance likely represent sets of correlated cytobands within the same genomic region, for which LASSO selects different cytobands across bootstrap iterations despite shared underlying biological signal.

medRxiv preprint doi: https://doi.org/10.64898/2026.06.24.26356446; this version posted July 1, 2026. The copyright holder for this preprint (which was not certified by peer review) is the author/funder, who has granted medRxiv a license to display the preprint in perpetuity.
It is made available under a CC-BY 4.0 International license .

Supplementary Fig. 11. Cytoband-level disease associations and mCA distribution across all chromosomes. a-w, Chromosome-level summary plots for chr1-7 and chr9-22 (**a-u**; chr8 data are shown in **Figure 6a**), chrX (**v**), and chrY (**w**). For each chromosome, top tracks display the number of ICD-10 disease chapters with significant cytoband-level associations (adjusted q<0.05) for gain (red), loss (blue), and CNLOH (orange). Bottom tracks show mCA distribution across the chromosome; each horizontal line represents an individual mCA event, with colors indicating CNV type (red, gain; blue, loss; orange, CNLOH). Chromosomal ideograms with cytoband annotations are shown at the bottom of each panel. For heterogeneous mCAs, cytobands were selected using LASSO-penalized Cox regression and refitted using standard Cox proportional hazards models adjusted for age, sex, and baseline disease burden. For homogeneous mCAs (12+, 14+, 15+, 18+, X-), which preclude cytoband-level resolution, standard Cox proportional hazards regression was performed comparing carriers against participants with no detectable mCA, adjusting for age, sex, and baseline disease burden, and the resulting chromosome-wide hazard ratio was applied uniformly across all cytobands of the affected chromosome. Multiple testing was corrected using the Benjamini-Hochberg method.

medRxiv preprint doi: https://doi.org/10.64898/2026.06.24.26356446; this version posted July 1, 2026. The copyright holder for this preprint (which was not certified by peer review) is the author/funder, who has granted medRxiv a license to display the preprint in perpetuity.
It is made available under a CC-BY 4.0 International license .

Supplementary Fig. 12. Cytoband-level disease associations by ICD-10 chapter. a-l, Volcano plots showing cytoband-level associations with incident disease risk across ICD-10 chapters: infectious diseases (**a**), hematologic disorders (**b**), endocrine/metabolic (**c**), mental/behavioral (**d**), nervous system (**e**), eye and ear (**f**), circulatory (**g**), respiratory (**h**), digestive (**i**), skin/subcutaneous (**j**), musculoskeletal (**k**), and genitourinary (**l**). The x-axis represents $\log_2$-transformed HRs and the y-axis represents $-\log_{10}$(FDR-adjusted q-values). Dot size reflects number of incident disease events among mCA carriers; colors indicate CNV type (red, gain; blue, loss; orange, CNLOH). Dashed vertical line indicates $\log_2$(HR)=0. Cytobands were selected using LASSO-penalized Cox regression and refitted using standard Cox proportional hazards models adjusted for age, sex, and baseline disease burden. Cytoband-level HRs were estimated as described in Methods. Multiple testing was corrected using the Benjamini-Hochberg method; only cytobands with adjusted q<0.05 are displayed.

medRxiv preprint doi: https://doi.org/10.64898/2026.06.24.26356446; this version posted July 1, 2026. The copyright holder for this preprint (which was not certified by peer review) is the author/funder, who has granted medRxiv a license to display the preprint in perpetuity.
It is made available under a CC-BY 4.0 International license .

Supplementary Fig. 13. Genome-wide circos plots of disease-associated cytobands by CNV type. a-c, Circos plots showing cytobands significantly associated with incident disease risk (adjusted q<0.05) for gain (**a**), loss (**b**), and CNLOH (**c**). Outer track displays chromosomal ideogram. Inner tracks show $\log_2$-transformed HRs for six selected ICD-10 disease chapters: infectious diseases (A+B), neoplasms (C), hematologic disorders (D), circulatory (I), respiratory (J), and genitourinary (N). Red bars indicate increased risk (HR>1); blue bars indicate decreased risk (HR<1). Cytobands were selected using LASSO-penalized Cox regression and refitted using standard Cox proportional hazards models adjusted for age, sex, and baseline disease burden. Multiple testing was corrected using the Benjamini-Hochberg method. Candidate genes within disease-associated cytobands (adjusted q<0.05) were retrieved from Ensembl GRCh37 using biomaRt. Protein-coding genes and microRNAs overlapping with significant cytoband coordinates are annotated.

medRxiv preprint doi: https://doi.org/10.64898/2026.06.24.26356446; this version posted July 1, 2026. The copyright holder for this preprint (which was not certified by peer review) is the author/funder, who has granted medRxiv a license to display the preprint in perpetuity.
It is made available under a CC-BY 4.0 International license .

Supplementary Fig. 14. Single-cell-resolved chromosome copy number analysis in bone marrow mononuclear cells from patients with myeloid diseases. a, Representative fluorescence in situ hybridization (FISH) images confirming trisomy 8 in BM cells from patients with myeloid neoplasms. Chr8 centromeric probes appear as red signals (Pt PDX, Pt 78, Pt 80, Pt 89) or aqua signals (Pt 202). Three discrete signals indicate trisomy 8. Nuclei are counterstained with DAPI (blue). **b**, Clinical and genetic profiles of patients with chr8 gain. Cytogenetics were assessed by conventional karyotyping and somatic mutations were identified by a 98-gene myeloid NGS panel. **c-h**, Numbat analysis of scRNA-seq data revealing CNVs in BM cells from each patient (see Methods). Columns represent chromosome locations (p arm on the left, q arm on the right) and rows represent individual cells. CNV states are shown as gain (red), bi-allelic gain (pink), loss (dark blue), bi-allelic loss (blue), and CNLOH (green). Left annotations indicate sample source (A, pre-treatment; B, post-treatment; C/D, relapse), cell type, and genotype cluster; distinct CNV groups were identified based on shared CNV patterns across cells.

medRxiv preprint doi: https://doi.org/10.64898/2026.06.24.26356446; this version posted July 1, 2026. The copyright holder for this preprint (which was not certified by peer review) is the author/funder, who has granted medRxiv a license to display the preprint in perpetuity.
It is made available under a CC-BY 4.0 International license .

Supplementary Fig. 15. Cytogenetic validation of trisomy 8 in patients with myeloid diseases. a-f, G-banding (trypsin-Giemsa) karyotype analysis was performed by Laboratory Corporation of America (Burlington, NC, USA). Nomenclature follows the International System for Human Cytogenomic Nomenclature (ISCN 2016). Each panel displays the diagnostic karyotype from an independent patient included in the scRNA-seq analysis shown in **Fig. 6** and **Supplementary Fig. 14**. Chr8 is highlighted with a dashed circle in each panel.

medRxiv preprint doi: https://doi.org/10.64898/2026.06.24.26356446; this version posted July 1, 2026. The copyright holder for this preprint (which was not certified by peer review) is the author/funder, who has granted medRxiv a license to display the preprint in perpetuity.
It is made available under a CC-BY 4.0 International license .

Supplementary Fig. 16. InferCNV analysis reveals focal transcriptional activation in aneuploid HSPCs. a, Genome-wide expression heatmap from InferCNV analysis of HSCs, CMPs, and GMPs in healthy donors (HD1, HD2, and HD3) serving as the reference population. Columns represent chromosomal positions (chromosomes 1–22), and rows represent individual cells. Color scale indicates modified expression values relative to the reference (blue, decreased expression; white, neutral; red, increased expression). **b**, InferCNV heatmap comparing cells with disomy 8 vs. cells with 8+ across multiple patients (Pt 78, Pt 80, Pt 89, Pt 202, Pt 297, and PDX). Hierarchical clustering groups cells by expression similarity. The prominent transcriptional upregulation (red box) is observed specifically on chr8 in 8+ cells but not in disomy 8 cells, with focal enrichment at the 8q21.3-24.23 region encompassing the *MYC* locus. **c**, InferCNV heatmap comparing cells with disomy 1 vs. cells with 1+ in two patients (Pt 74 with 1q gain and Pt 187 with whole chr1 gain). NA indicates cells with undetermined copy number status. Transcriptional activation is focally enriched within the 1q21.1–23.2 region (red box), which contains the *S100A1-S100A16* gene cluster, including *S100A9*. **d-e**, Quantitative analysis of chr8 (**d**) and chr1 (**e**) gene expression in HSCs across genomic positions. Smoothed LOESS curves show mean InferCNV-adjusted expression levels for healthy donors (HD, blue), disomy cells (orange), and individual samples with 8+ or 1+ (colored lines). Each dot represents a single gene's mean expression. Green vertical lines mark key disease-associated loci: *MYC* at 8q24.21 and *S100A9* at 1q21.3. Gray dashed lines indicate centromere positions. Bottom panels show chromosome ideograms with cytoband annotations. 8+ cells demonstrate elevated expression across chr8, particularly in the 8q21.3–24.23 region, and 1+ cells show elevated expression predominantly on 1q, particularly in the 1q21.1–23.2 region, consistent with the focal amplification patterns

medRxiv preprint doi: https://doi.org/10.64898/2026.06.24.26356446; this version posted July 1, 2026. The copyright holder for this preprint (which was not certified by peer review) is the author/funder, who has granted medRxiv a license to display the preprint in perpetuity.
It is made available under a CC-BY 4.0 International license .

observed in panels **b** and **c**. For panels **b-e**, copy number status was determined using Numbat, and InferCNV was used for visualization of transcriptional effects.

medRxiv preprint doi: https://doi.org/10.64898/2026.06.24.26356446; this version posted July 1, 2026. The copyright holder for this preprint (which was not certified by peer review) is the author/funder, who has granted medRxiv a license to display the preprint in perpetuity.
It is made available under a CC-BY 4.0 International license .

Supplementary Fig. 17. Genomic landscape of myeloid diseases with trisomy 8 and functional interrogation of MYC overexpression. a, Oncoplot showing somatic mutations and chromosomal alterations in patients with myeloid diseases. Columns represent individual patients; rows represent genes or chromosomal alterations. Top bar shows number of alterations per patient. Right bar shows mutation or mCA frequency (%). Patients are grouped by disease type: acute myeloid leukemia (AML; n=135), triple-negative myelofibrosis (TN-MF; n=46), and myelodysplastic syndrome (MDS; n=36). Blue indicates presence of alteration. **b**, Representative MYC immunohistochemistry (IHC) staining in BM samples from patients with disomy 8 vs. trisomy 8 across disease types (scale bar, 50 μm). **c**, Quantification of MYC IHC staining comparing disomy 8 (n=153) vs. trisomy 8 (n=39) among patients with available IHC data (n=192/217); P value calculated using Wilcoxon rank-sum test. **d**, Schematic of the Mx1-Cre$^{+/-}$;Rosa26$^{LSL\text{-}MYC/LSL\text{-}MYC}$ mouse model for conditional *MYC* overexpression following pIpC induction. **e-f**, Confirmation of MYC overexpression by qRT-PCR (**e**) and immunoblotting (**f**). **g-i**, Flow cytometric analysis of BM populations in WT vs. MYC mice: HSC/MPP (% of Lin-) (**g**), CMP/GMP/MEP (% of Lin-) (**h**), and Gr1+/CD11b+ myeloid cells (% of total) (**i**). Violin plots with embedded box plots show median and interquartile range; P values were calculated using Wilcoxon rank-sum test. **j**, Box plots showing raw CRP levels (mg/L) across three groups: no mCA (n=329,762), 8+ with MCF <10% (n=73), and 8+ with MCF ≥10% (n=54). Boxes represent the interquartile range (IQR, 25th-75th percentiles), with the horizontal line indicating the median. Whiskers extend to 1.5×IQR. Outliers beyond the 95th percentile are not displayed for visualization purposes. **k,** Violin plots showing the distribution of log-transformed CRP [log(CRP+1)] for the same groups. The width of each violin represents the kernel density estimate of the data

medRxiv preprint doi: https://doi.org/10.64898/2026.06.24.26356446; this version posted July 1, 2026. The copyright holder for this preprint (which was not certified by peer review) is the author/funder, who has granted medRxiv a license to display the preprint in perpetuity.
It is made available under a CC-BY 4.0 International license .

distribution, with embedded box plots displaying the median and IQR. In **j** and **k**, group comparisons were performed using the Wilcoxon rank-sum test for raw CRP values and Welch's t-test for log-transformed CRP. Individuals with 8+ and MCF ≥10% had significantly elevated CRP compared to no-mCA controls [median 1.84 (IQR 1.43-2.54) vs. 1.60 (IQR 1.30-2.07) mg/L; Wilcoxon p=0.012]. In contrast, 8+ with MCF <10% showed no significant difference in CRP compared to controls [median 1.57 (IQR 1.27-2.09) mg/L; Wilcoxon p=0.598]. **l,** Scatter plot showing the relationship between 8+ MCF and log-transformed CRP [log(CRP+1)]. Individuals without mCA are shown at MCF=0 (blue; n=5,000 randomly sampled for visualization), and 8+ carriers are shown at their actual MCF values (red; n=127). The dashed line represents the linear regression fit with 95% CI (shaded area). Multivariable linear regression was used to assess the association between MCF and log-transformed CRP, adjusted for age and sex. A significant positive association was observed (β=0.164, 95% CI: 0.024-0.304, p=0.021), indicating that higher 8+ clone burden is associated with increased levels of systemic inflammation. **m-n**, Box plots showing circulating TNFα (**m**) and IL6 (**n**) protein levels measured by Olink proteomics in no mCA controls (n=41,962 for TNFα; n=42,601 for IL6) vs. 8+ carriers (n=16). Values are expressed as Normalized Protein eXpression (NPX, log2 scale). Boxes represent IQR, horizontal lines indicate median, and whiskers extend to 1.5×IQR. P values were calculated using Wilcoxon rank-sum test.

medRxiv preprint doi: https://doi.org/10.64898/2026.06.24.26356446; this version posted July 1, 2026. The copyright holder for this preprint (which was not certified by peer review) is the author/funder, who has granted medRxiv a license to display the preprint in perpetuity.
It is made available under a CC-BY 4.0 International license .

Supplementary Fig. 18. Functional validation of *S100A9* as a candidate gene in trisomy 1. a-b, Numbat analysis of scRNA-seq data revealing CNVs in BM cells from patients with 1+: Pt 74 (**a**) and Pt 187 (**b**). Columns represent chromosome locations (p arm on the left, q arm on the right) and rows represent individual cells. CNV states are shown as gain (red), biallelic gain (pink), loss (blue), and CNLOH (green). Left annotations indicate sample source (first column), cell type (cluster annotation; second column), and genotype cluster (third column). **c**, Differentially expressed genes in HSCs from patients with 1+ compared to healthy donors (HD). scRNA-seq was performed on 25,932 cells (1+, n=2,372; disomy 1, n=23,560; classified using Numbat, see Methods) from 4 samples (2 patients), and 16,504 cells from 3 HD. Dot size reflects fraction of 1+ cells expressing each gene; colors indicate upregulation (red) or downregulation (blue). **d**, Chr1 genes ranked by log_2 fold change in 1+ HSCs vs. HD. **e-f**, Confirmation of S100A9 overexpression by qRT-PCR (**e**) and immunoblotting (**f**) in spleen (SP) from S100A9 transgenic (S100A9Tg) vs. wild type (WT) mice. S100A9 expression levels in S100A9Tg mice are comparable to those observed in 1+ human HSPCs (panel **d**). **g-h**, Peripheral blood monocyte counts (**g**) and RBC counts (**h**) in S100A9Tg mice vs. WT controls. **i-j**, Monocyte counts (**i**) and RBC counts (**j**) in UKBB participants with 1+ vs. non-carriers. **k-l**, Overall survival in S100A9Tg (n=59) vs. WT (n=25) mice (**k**) and in UKBB participants with 1+ (n=67) vs. non-carriers (n=410,204) (**l**). **m**, Histological analysis of fibrosis using reticulin staining in lung, heart, colon, liver, and spleen from S100A9Tg vs. WT mice; original magnification x200; scale bar, 50μm. For **g-j**, violin plots with embedded box plots show median and interquartile range. For **e** and **g-j**, P values were calculated using the Wilcoxon rank-sum test. For **k** and **l**, shaded areas represent 95% CIs; P values were calculated using the log-rank test.

medRxiv preprint doi: https://doi.org/10.64898/2026.06.24.26356446; this version posted July 1, 2026. The copyright holder for this preprint (which was not certified by peer review) is the author/funder, who has granted medRxiv a license to display the preprint in perpetuity.
It is made available under a CC-BY 4.0 International license .

Supplementary Fig. 19. Levels of pro-inflammatory cytokines and alarmins in 1+ carriers. a-f, Box plots showing circulating protein levels measured by Olink proteomics in no mCA controls vs. 1+ carriers. Sample sizes for 1+ carriers: n=6 for all cytokines and alarmins except TLR4. Values are expressed as Normalized Protein eXpression (NPX, log2 scale). Boxes represent the IQR, horizontal lines indicate the median, and whiskers extend to 1.5×IQR. P values were calculated using the Wilcoxon rank-sum test. IL1β (p=0.0262) (**a**), TNFα (p=0.0347) (**b**), and S100A11 (p=0.0447) (**c**) showed significantly elevated levels in 1+ carriers, while S100A12 (p=0.0846) (d), TLR4 (p=0.226) (e), and IL6 (p=0.506) (f) were numerically higher but did not reach statistical significance. Note that the *S100A11* and *S100A12* genes are located on chromosome 1q21.3, which is associated with increased risk of multimorbidity (see **Supplementary Fig. 11A** and **Supplementary Fig. 12A**).

Supplementary Fig. 1

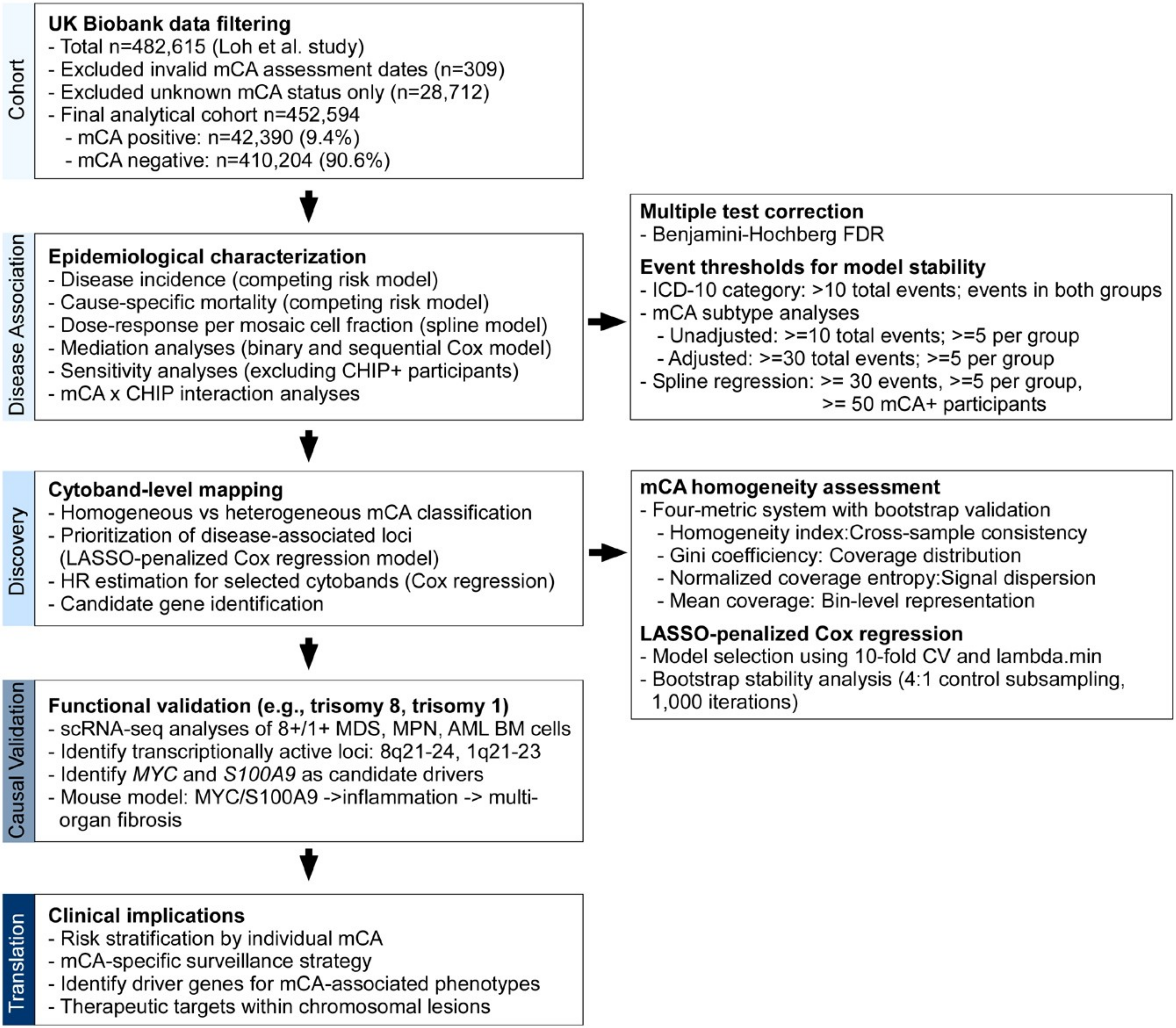

Supplementary Fig. 2

medRxiv preprint doi: https://doi.org/10.64898/2026.06.24.26356446; this version posted July 1, 2026. The copyright holder for this preprint (which was not certified by peer review) is the author/funder, who has granted medRxiv a license to display the preprint in perpetuity.
It is made available under a CC-BY 4.0 International license .

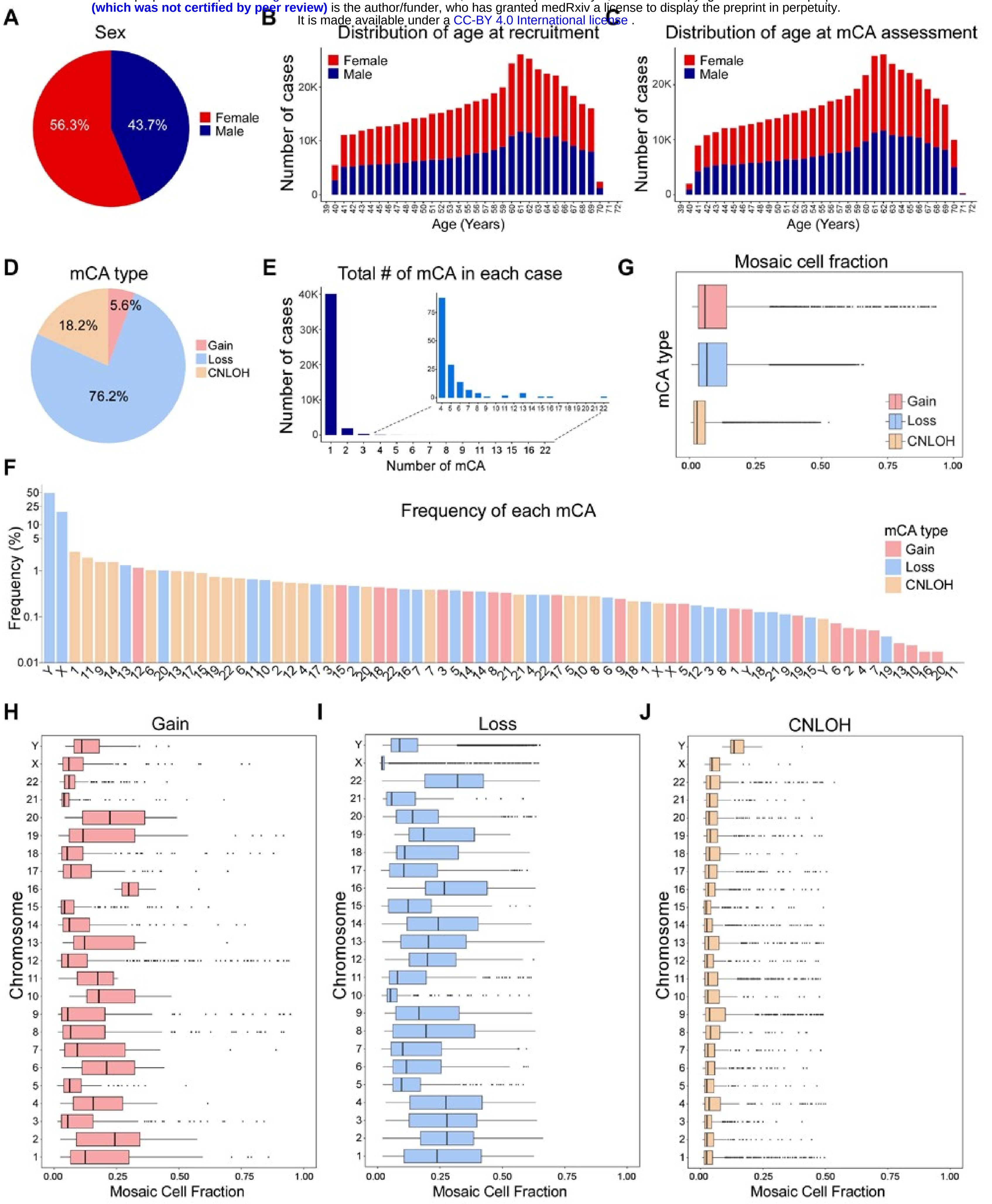

Supplementary Fig. 3

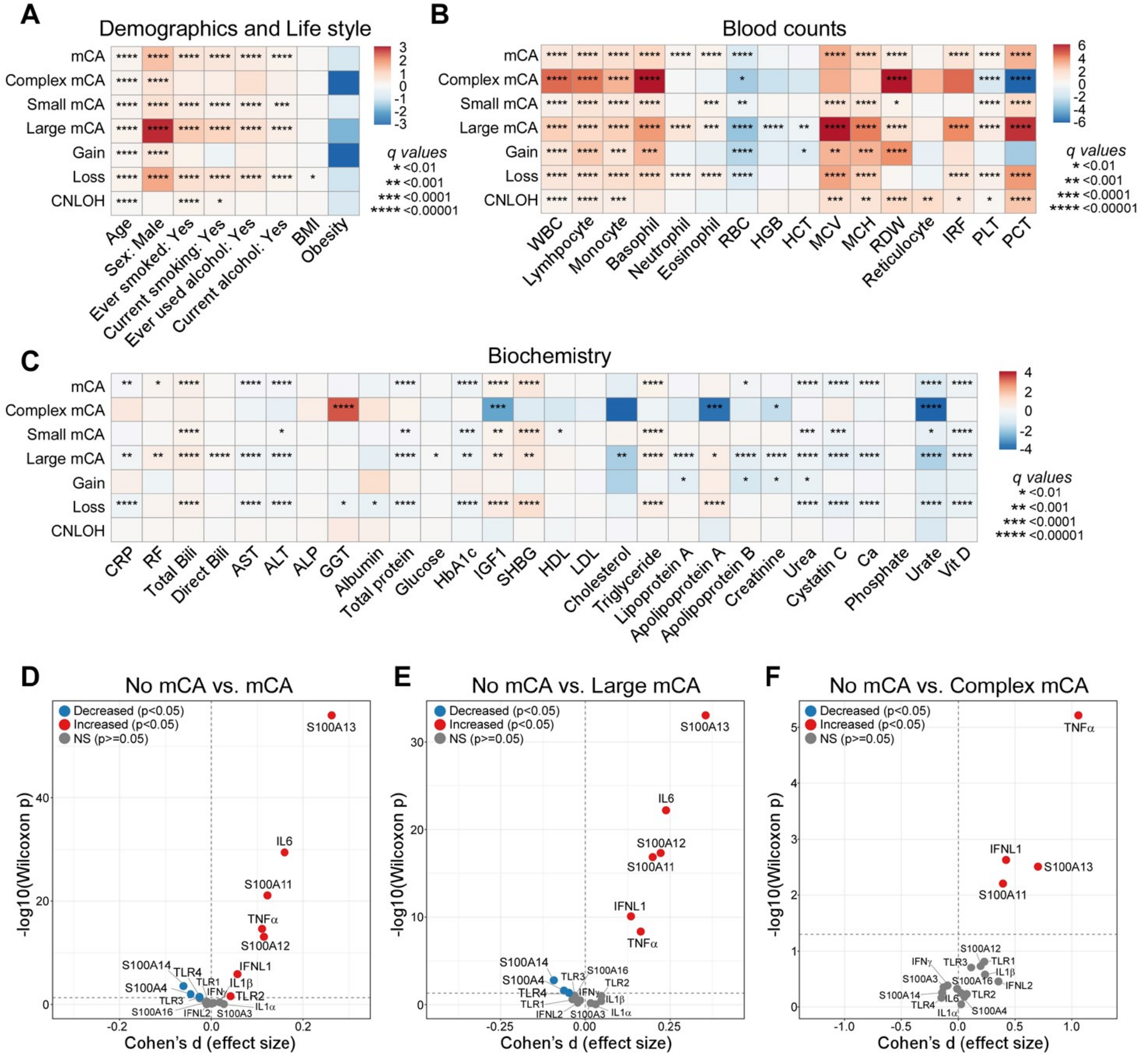

Supplementary Fig. 4

medRxiv preprint doi: https://doi.org/10.64898/2026.06.24.26356446; this version posted July 1, 2026. The copyright holder for this preprint (which was not certified by peer review) is the author/funder, who has granted medRxiv a license to display the preprint in perpetuity.
It is made available under a CC-BY 4.0 International license .

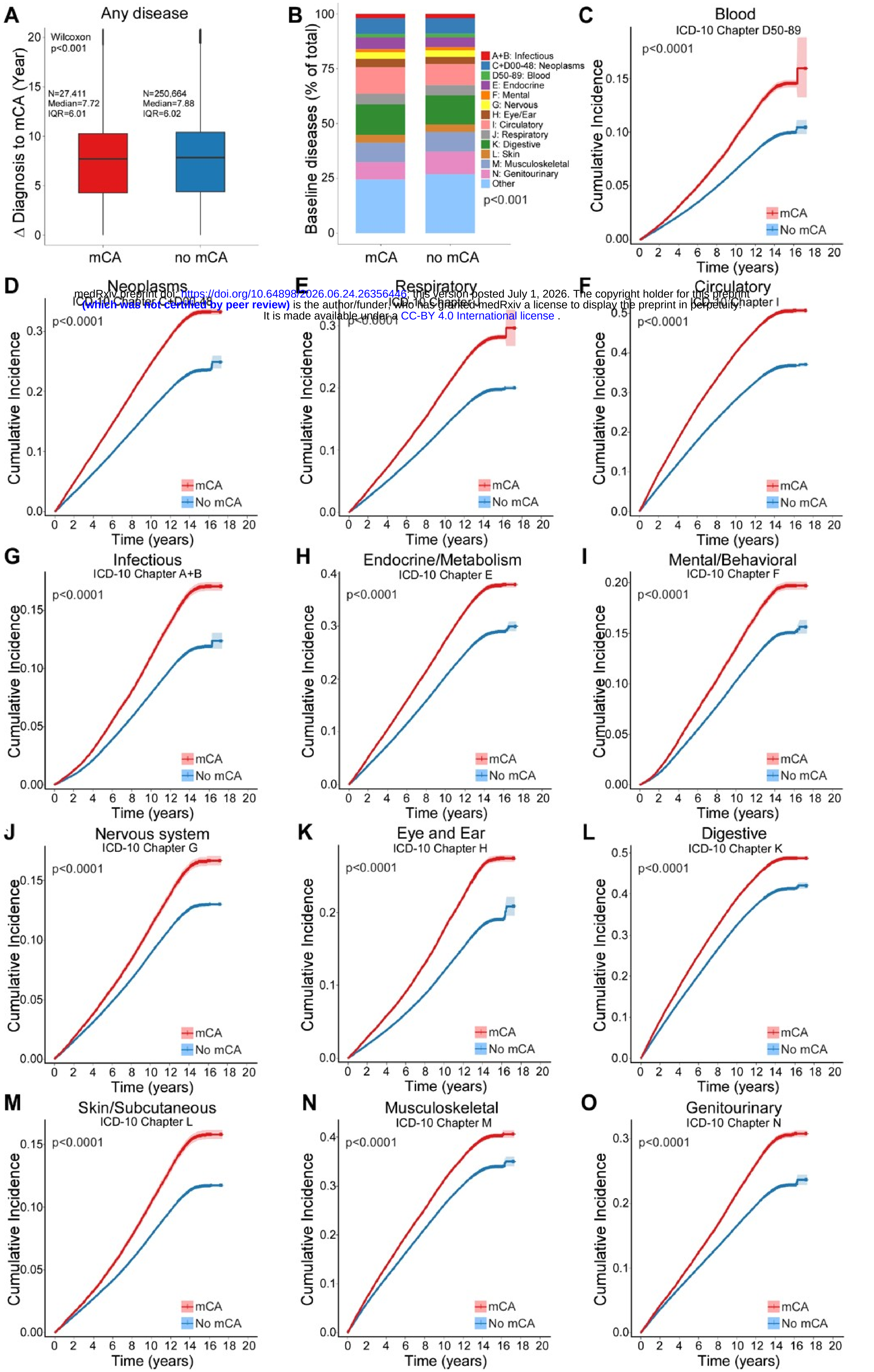

Supplementary Fig. 5

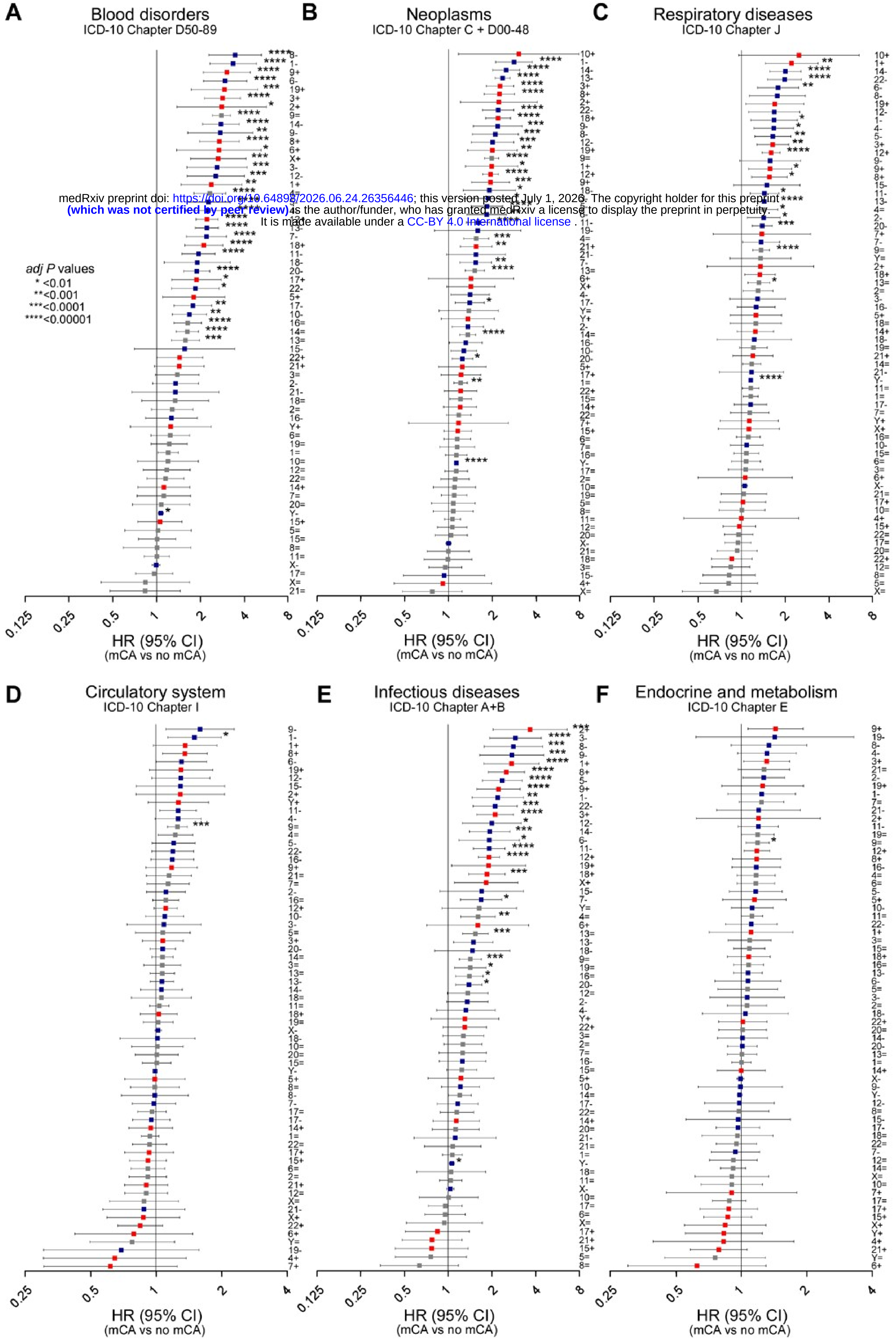

medRxiv preprint doi: https://doi.org/10.64898/2026.06.24.26356446; this version posted July 1, 2026. The copyright holder for this preprint (which was not certified by peer review) is the author/funder, who has granted medRxiv a license to display the preprint in perpetuity.
It is made available under a CC-BY 4.0 International license .

medRxiv preprint doi: https://doi.org/10.64898/2026.06.24.26356446; this version posted July 1, 2026. The copyright holder for this preprint
(which was not certified by peer review) is the author/funder, who has granted medRxiv a license to display the preprint in perpetuity.
It is made available under a CC-BY 4.0 International license .

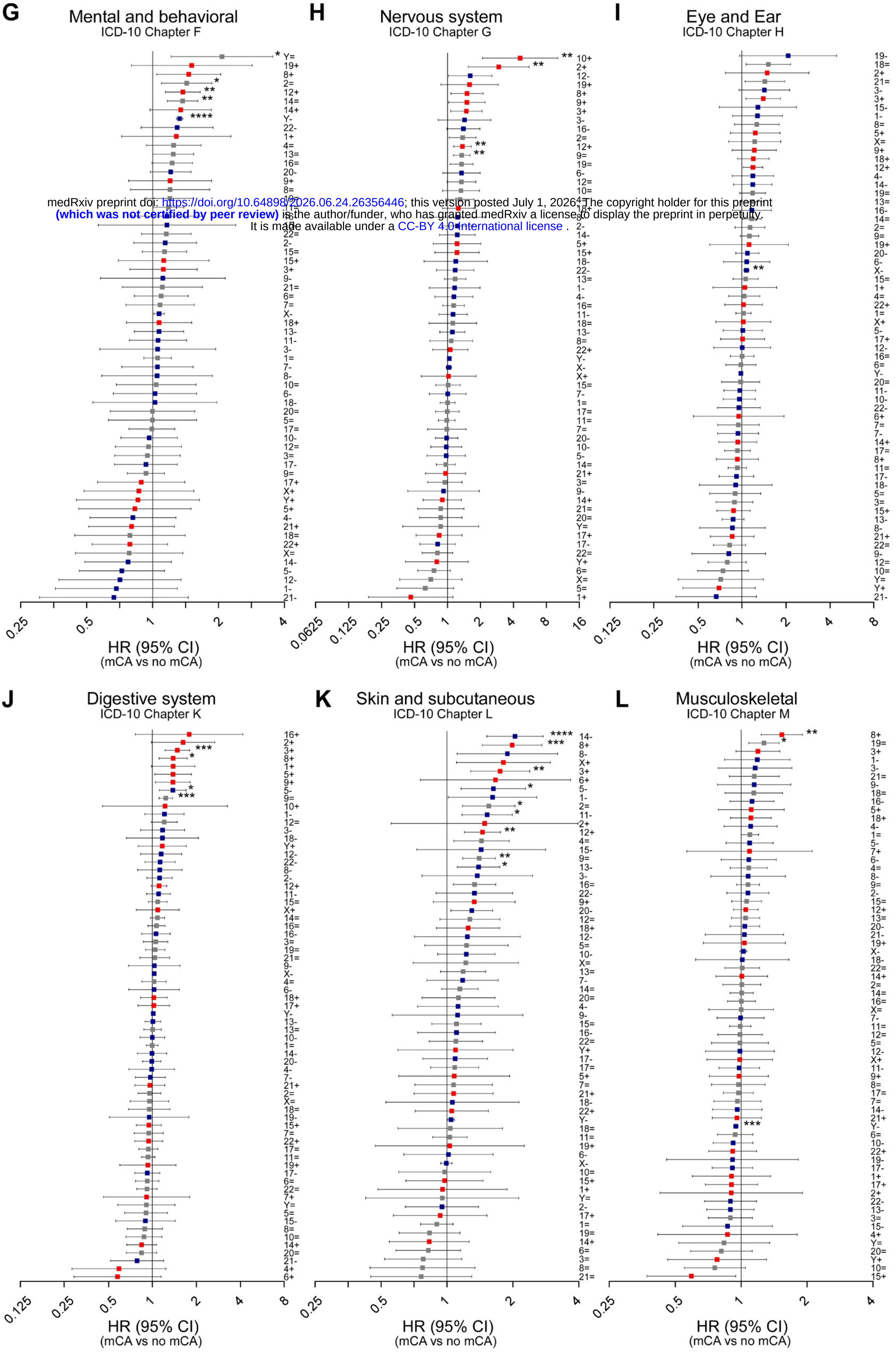

M

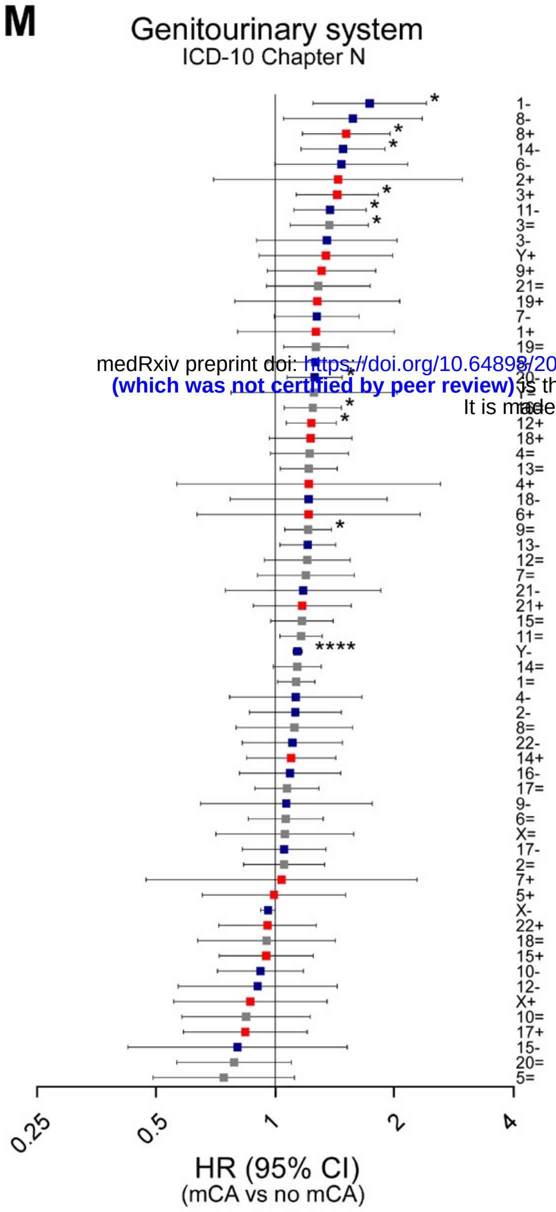

medRxiv preprint doi: https://doi.org/10.64898/2026.06.24.26356446; this version posted July 1, 2026. The copyright holder for this preprint (which was not certified by peer review) is the author/funder, who has granted medRxiv a license to display the preprint in perpetuity.
It is made available under a CC-BY 4.0 International license .

medRxiv preprint doi: https://doi.org/10.64898/2026.06.24.26356446; this version posted July 1, 2026. The copyright holder for this preprint (which was not certified by peer review) is the author/funder, who has granted medRxiv a license to display the preprint in perpetuity.
It is made available under a CC-BY 4.0 International license .

Supplementary Fig. 6

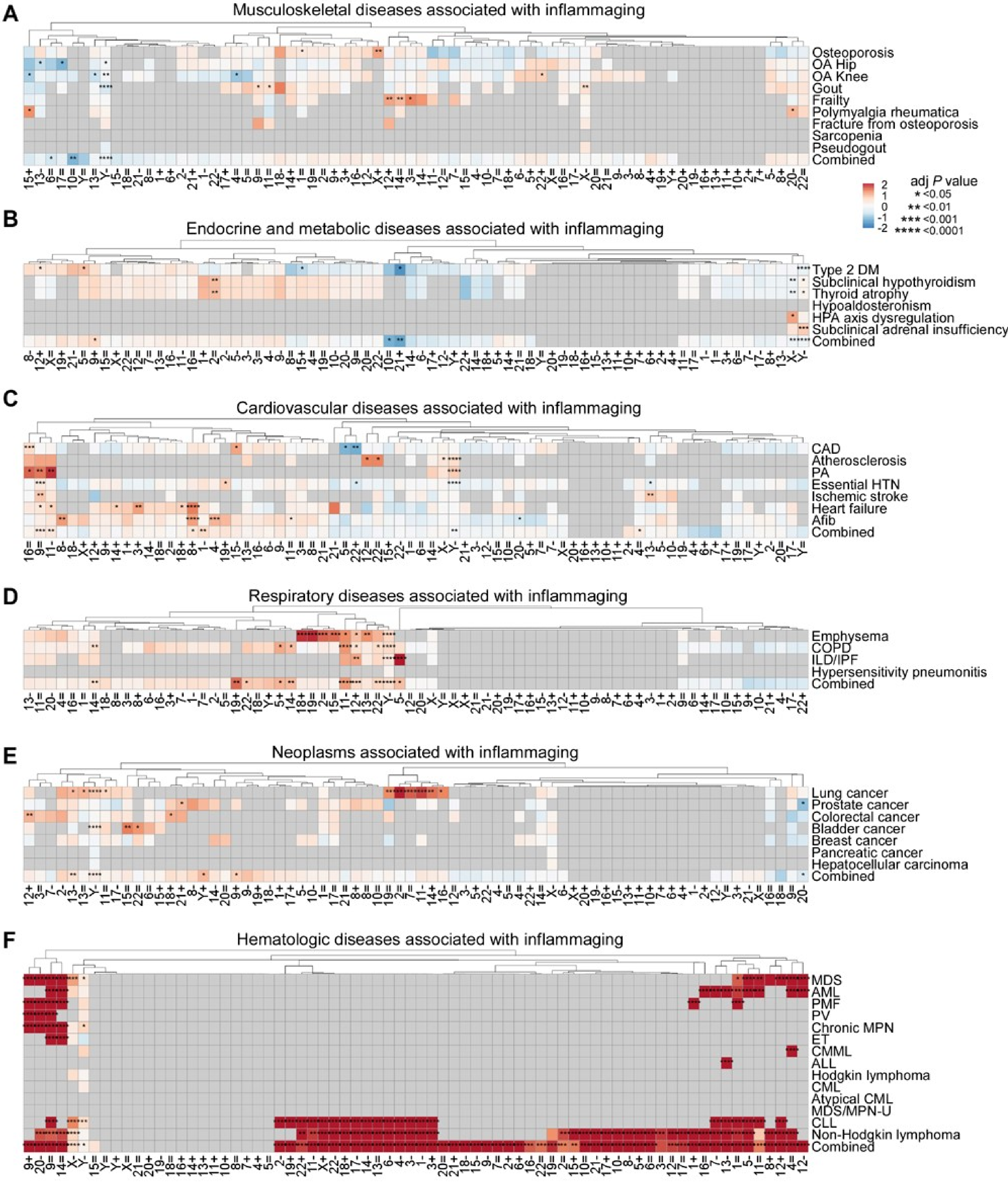

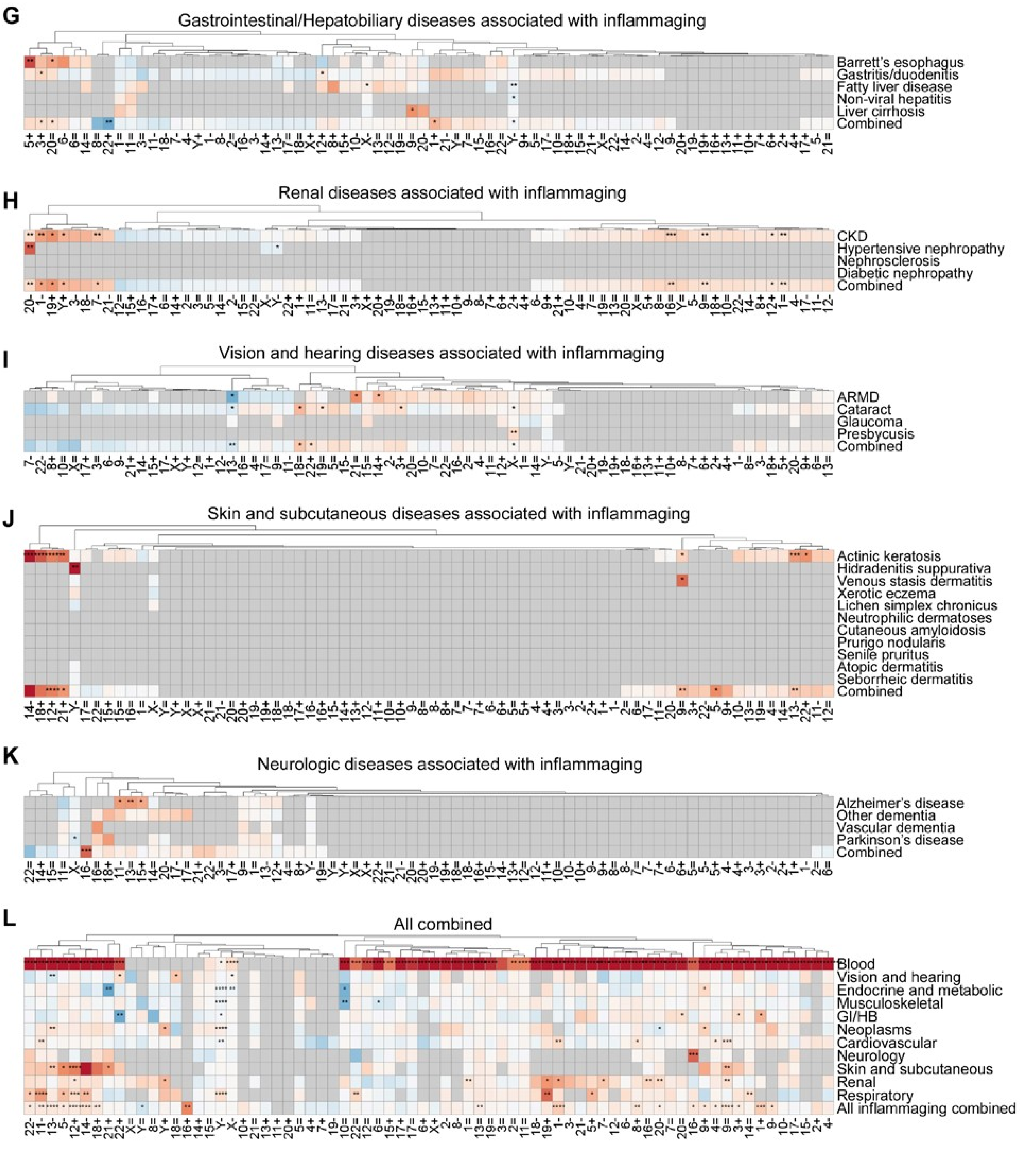
G
Gastrointestinal/Hepatobiliary diseases associated with inflammaging
Barrett's esophagus
Gastritis/duodenitis
Fatty liver disease
Non-viral hepatitis
Liver cirrhosis
Combined
H
Renal diseases associated with inflammaging
CKD
Hypertensive nephropathy
Nephrosclerosis
Diabetic nephropathy
Combined
I
Vision and hearing diseases associated with inflammaging
ARMD
Cataract
Glaucoma
Presbycusis
Combined
J
Skin and subcutaneous diseases associated with inflammaging
Actinic keratosis
Hidradenitis suppurativa
Venous stasis dermatitis
Xerotic eczema
Lichen simplex chronicus
Neutrophilic dermatoses
Cutaneous amyloidosis
Prurigo nodularis
Senile pruritus
Atopic dermatitis
Seborrheic dermatitis
Combined
K
Neurologic diseases associated with inflammaging
Alzheimer's disease
Other dementia
Vascular dementia
Parkinson's disease
Combined
L
All combined
Blood
Vision and hearing
Endocrine and metabolic
Musculoskeletal
GI/HB
Neoplasms
Cardiovascular
Neurology
Skin and subcutaneous
Renal
Respiratory
All inflammaging combined

Supplementary Fig. 7

medRxiv preprint doi: https://doi.org/10.64898/2026.06.24.26356446; this version posted July 1, 2026. The copyright holder for this preprint (which was not certified by peer review) is the author/funder, who has granted medRxiv a license to display the preprint in perpetuity.
It is made available under a CC-BY 4.0 International license .

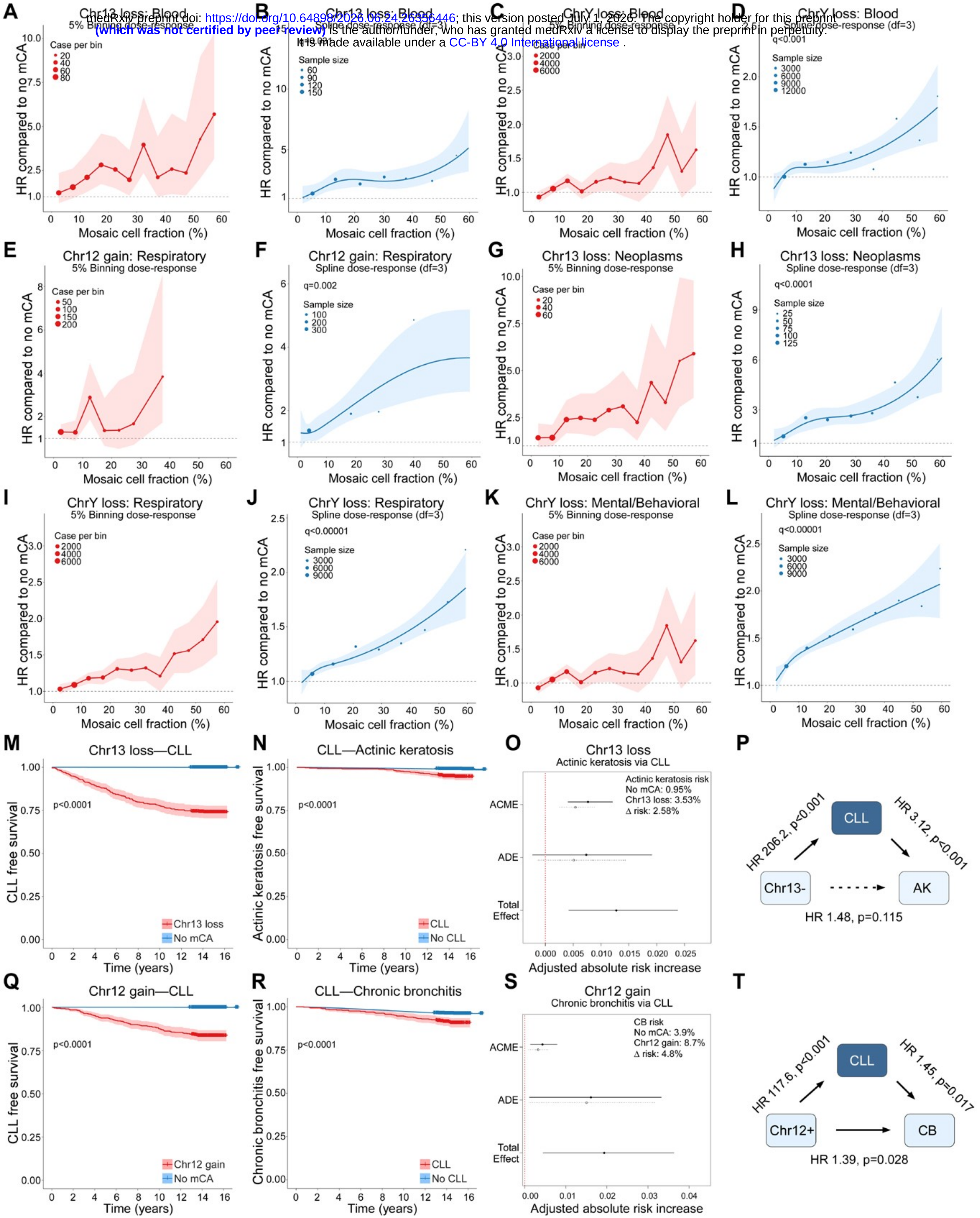

Supplementary Fig. 8

medRxiv preprint doi: https://doi.org/10.64898/2026.06.24.26356446; this version posted July 1, 2026. The copyright holder for this preprint (which was not certified by peer review) is the author/funder, who has granted medRxiv a license to display the preprint in perpetuity.
It is made available under a CC-BY 4.0 International license .

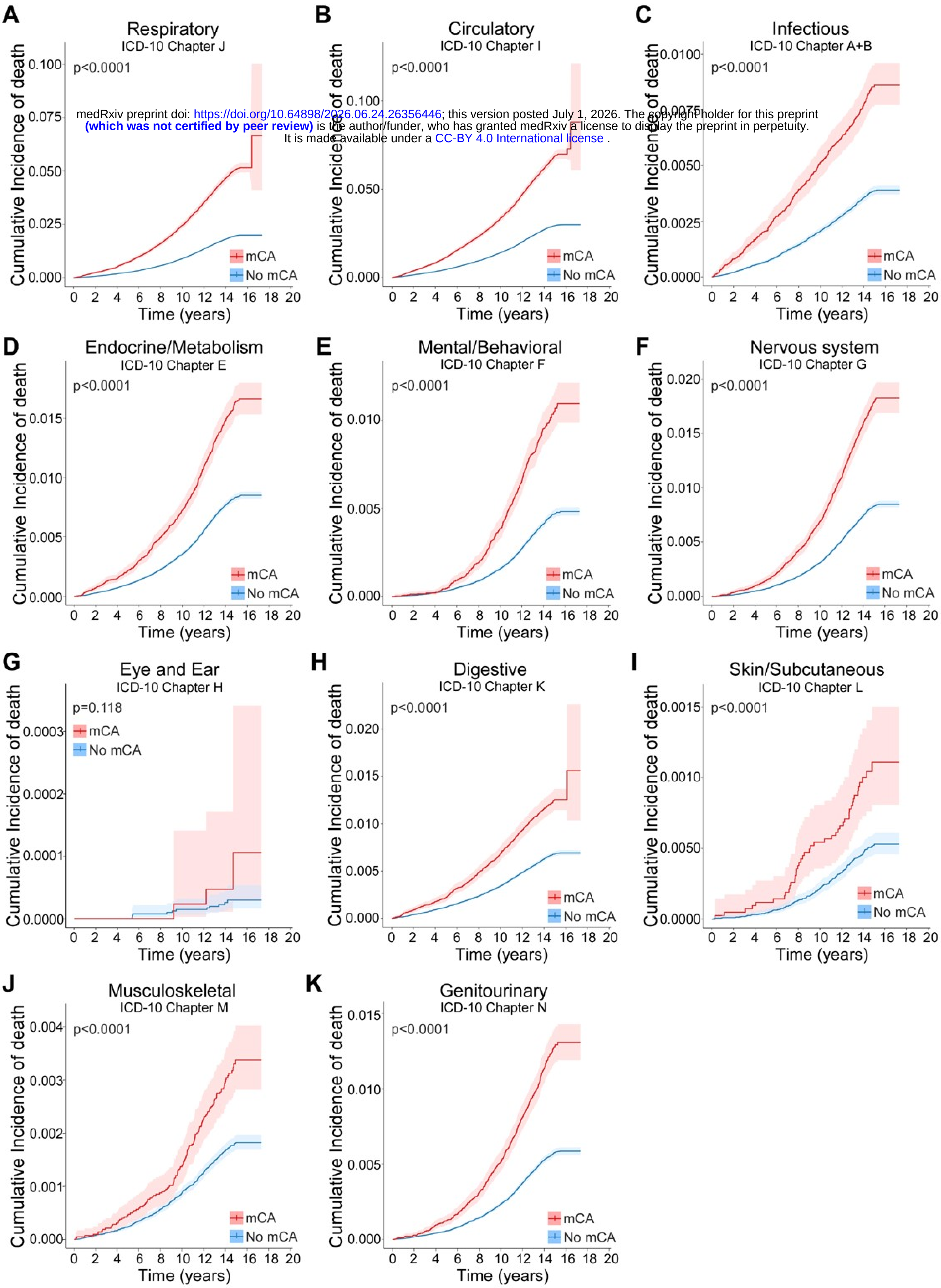

Supplementary Fig. 9

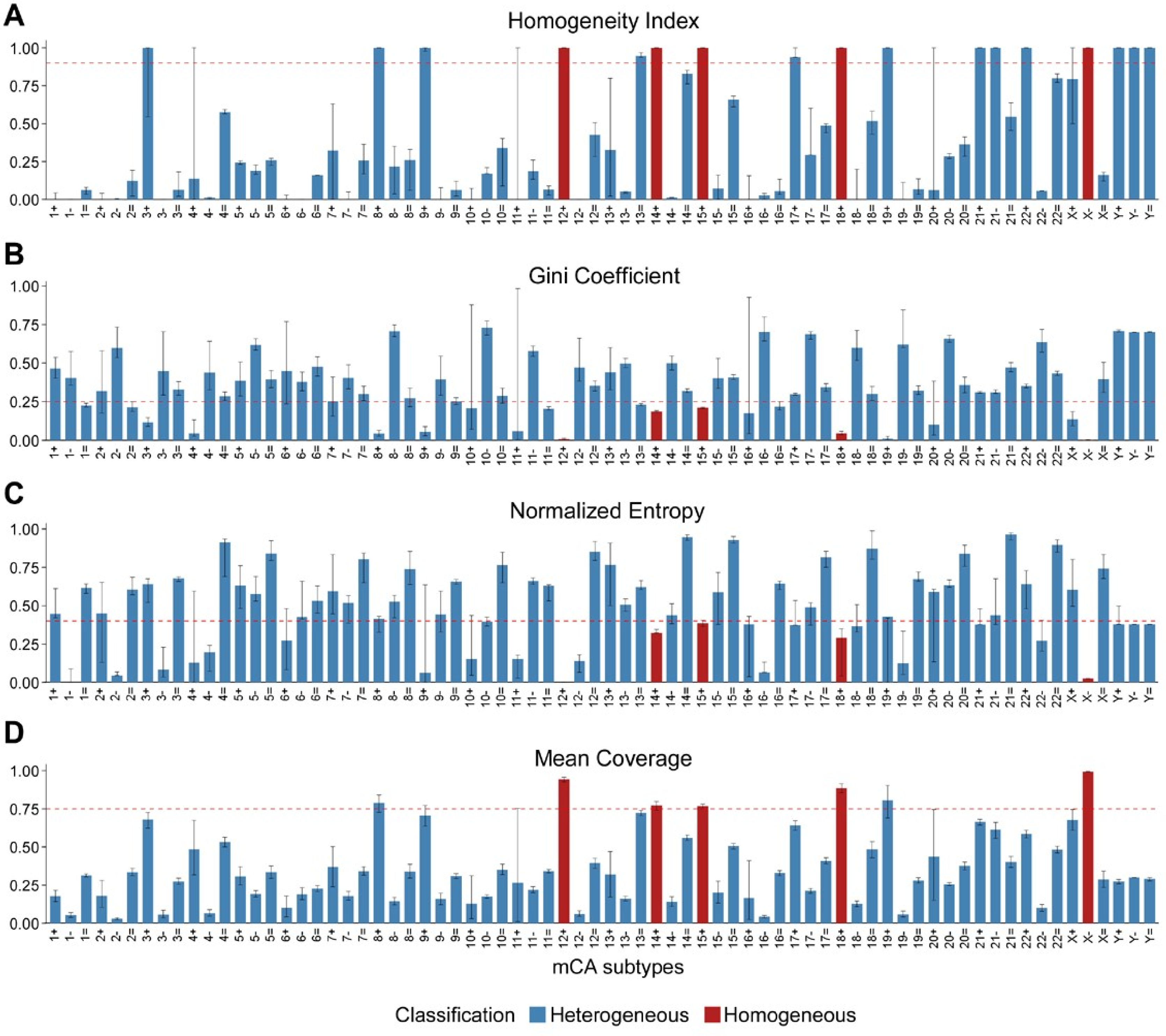

Supplementary Fig. 10

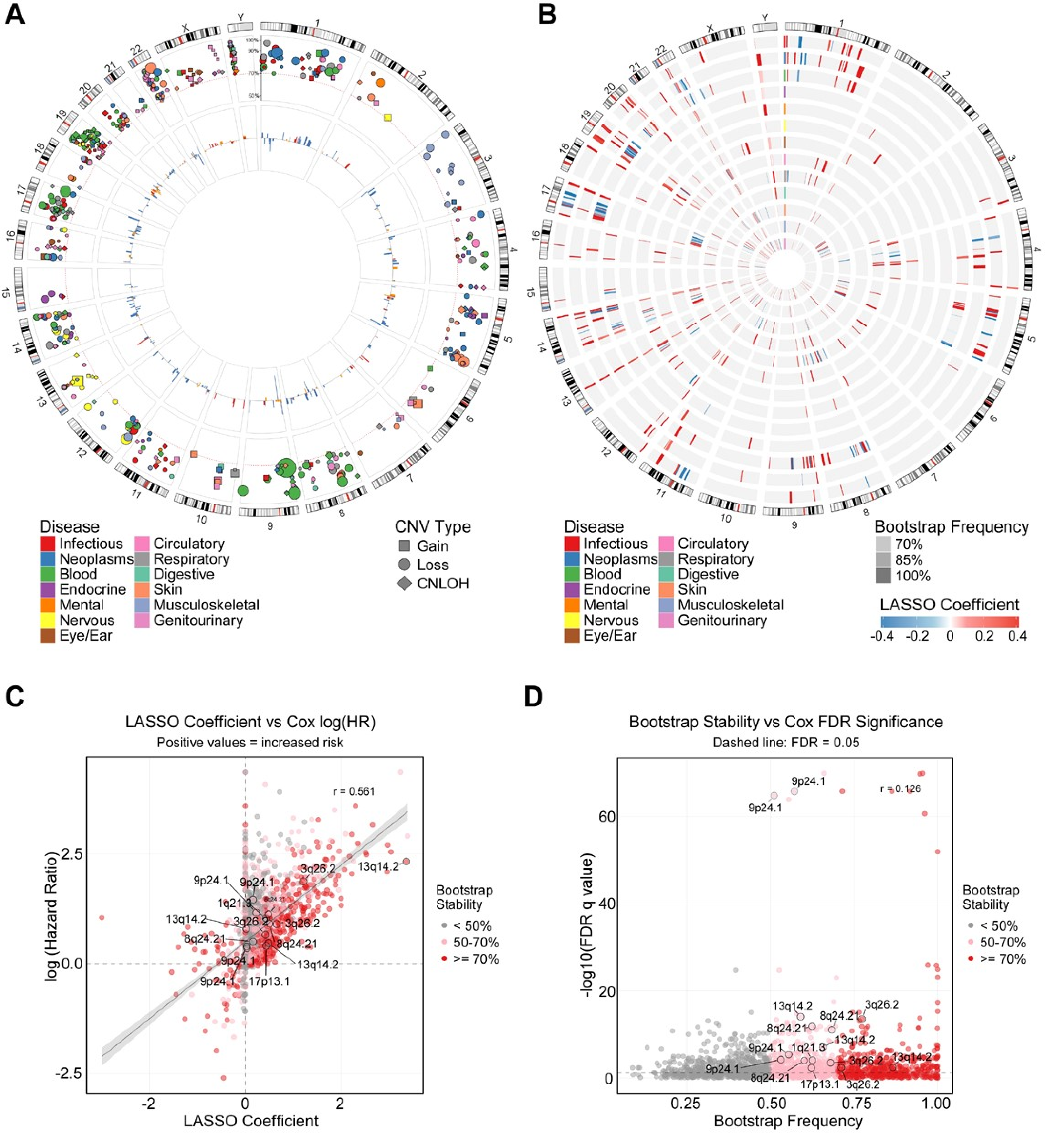

Supplementary Fig. 11

medRxiv preprint doi: https://doi.org/10.64898/2026.06.24.26356446; this version posted July 1, 2026. The copyright holder for this preprint
(which was not certified by peer review) is the author/funder, who has granted medRxiv a license to display the preprint in perpetuity.
It is made available under a CC-BY 4.0 International license .

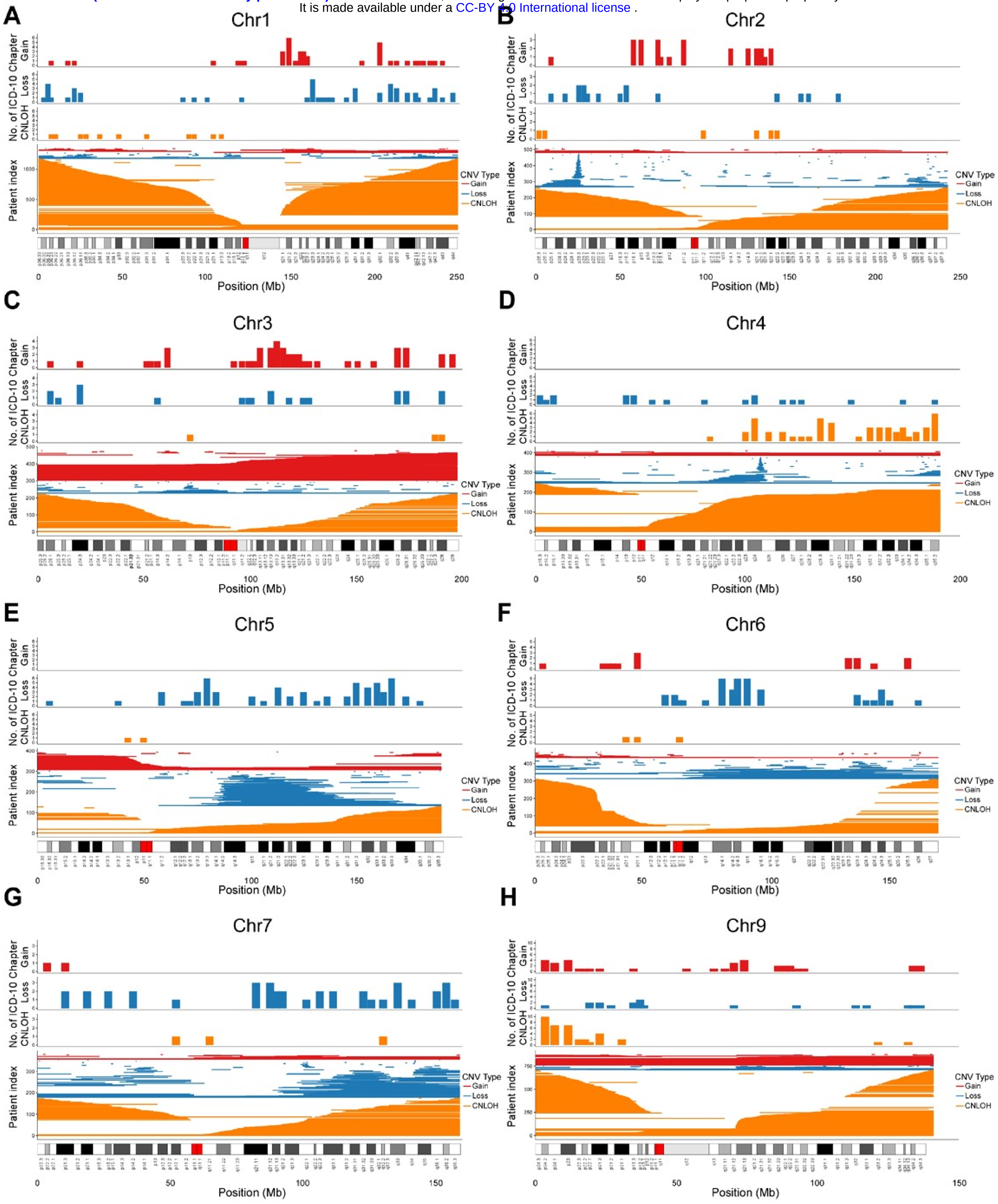

medRxiv preprint doi: https://doi.org/10.64898/2026.06.24.26356446; this version posted July 1, 2026. The copyright holder for this preprint
(which was not certified by peer review) is the author/funder, who has granted medRxiv a license to display the preprint in perpetuity.
It is made available under a CC-BY 4.0 International license .

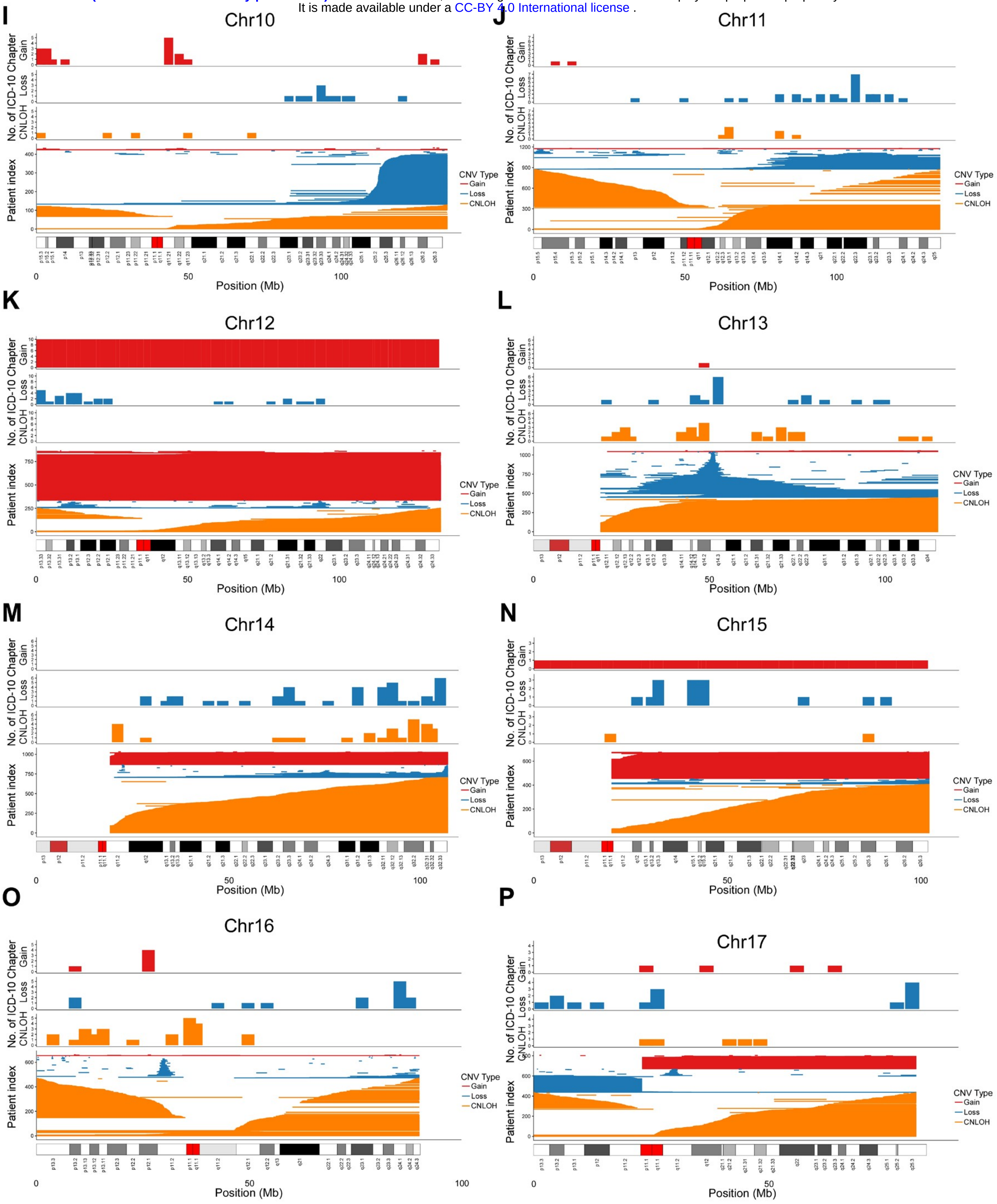

medRxiv preprint doi: https://doi.org/10.64898/2026.06.24.26356446; this version posted July 1, 2026. The copyright holder for this preprint
(which was not certified by peer review) is the author/funder, who has granted medRxiv a license to display the preprint in perpetuity.
It is made available under a CC-BY 4.0 International license .

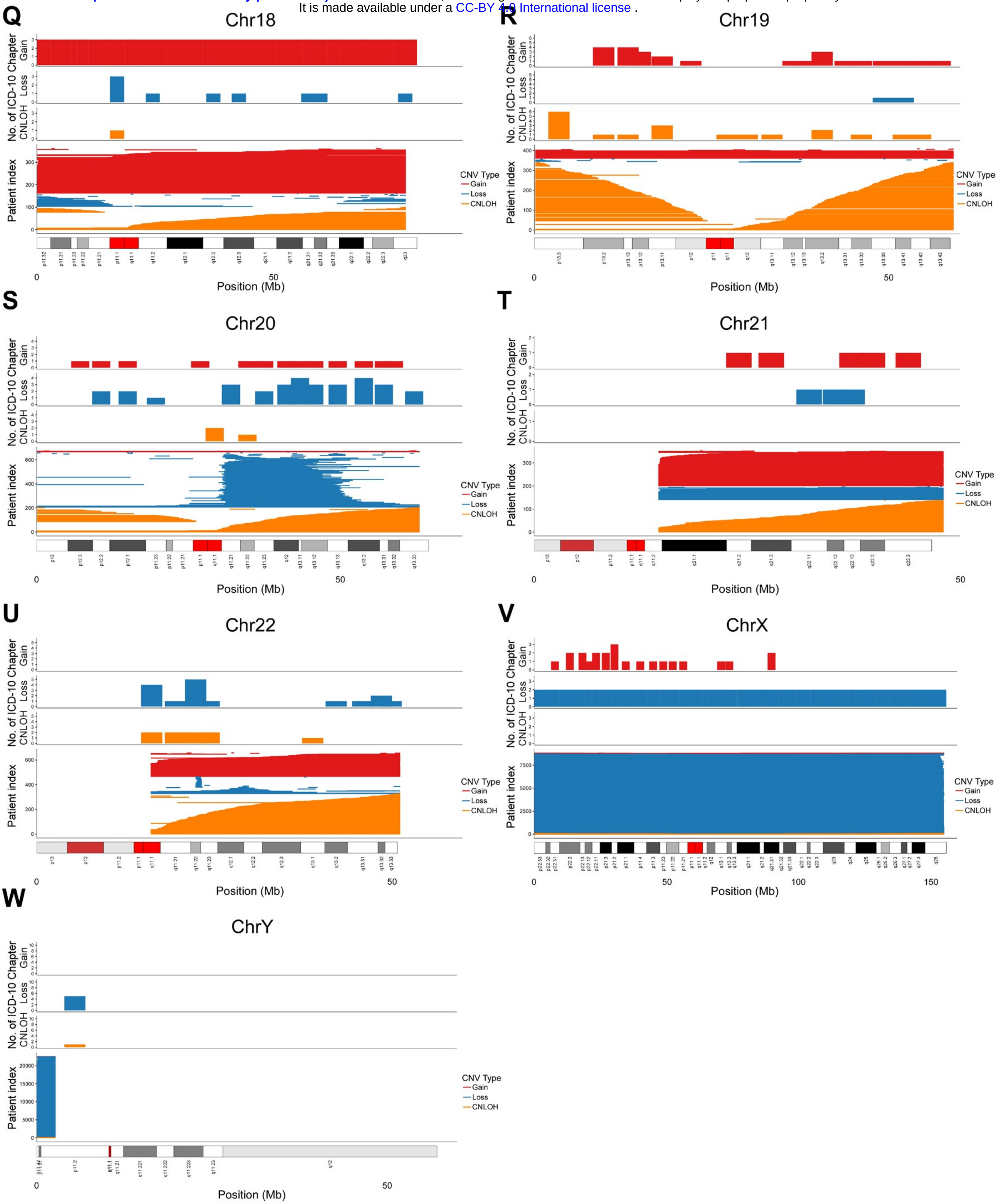

Supplementary Fig. 12

medRxiv preprint doi: https://doi.org/10.64898/2026.06.24.26356446; this version posted July 1, 2026. The copyright holder for this preprint (which was not certified by peer review) is the author/funder, who has granted medRxiv a license to display the preprint in perpetuity.
It is made available under a CC-BY 4.0 International license .

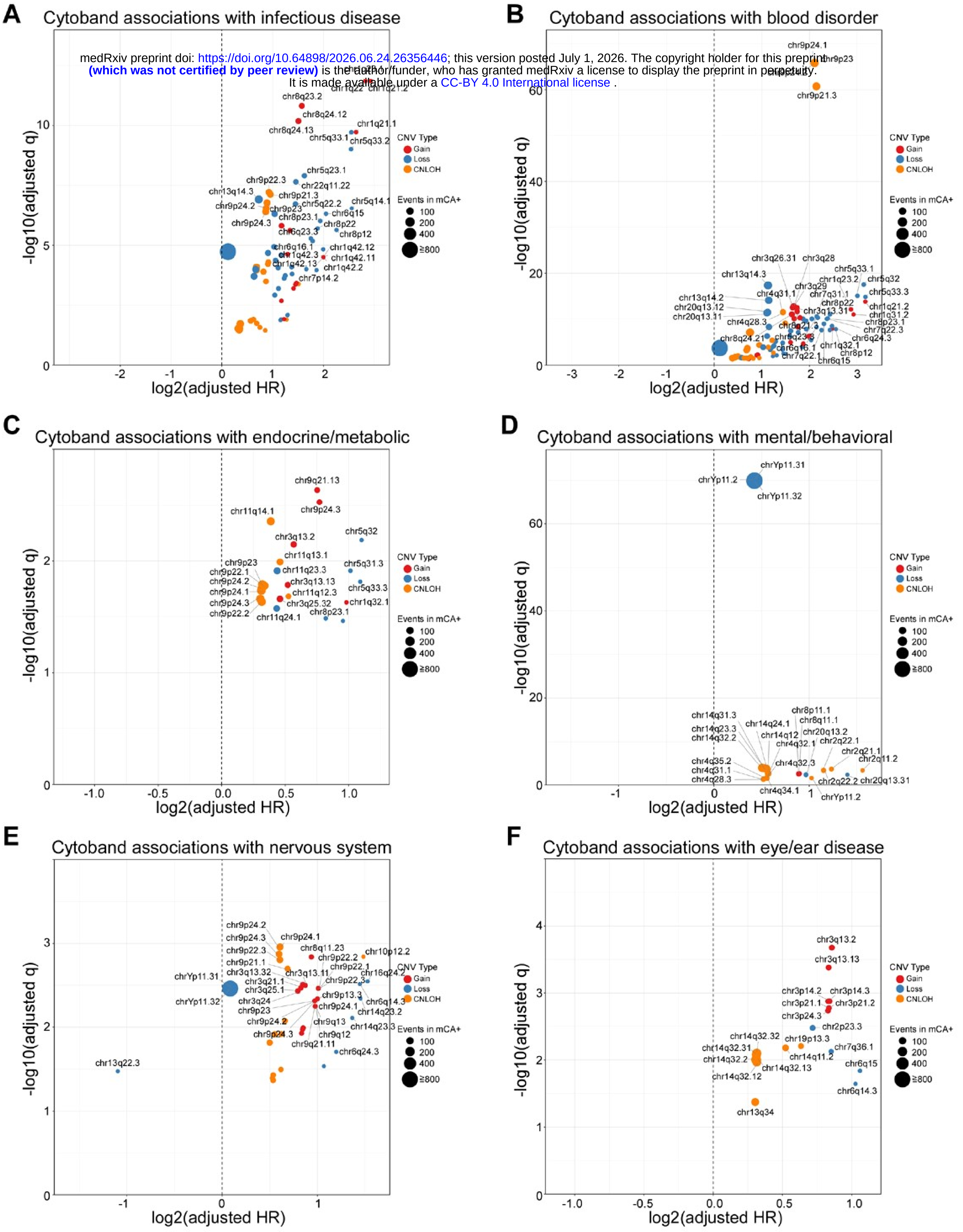

medRxiv preprint doi: https://doi.org/10.64898/2026.06.24.26356446; this version posted July 1, 2026. The copyright holder for this preprint (which was not certified by peer review) is the author/funder, who has granted medRxiv a license to display the preprint in perpetuity.
It is made available under a CC-BY 4.0 International license .

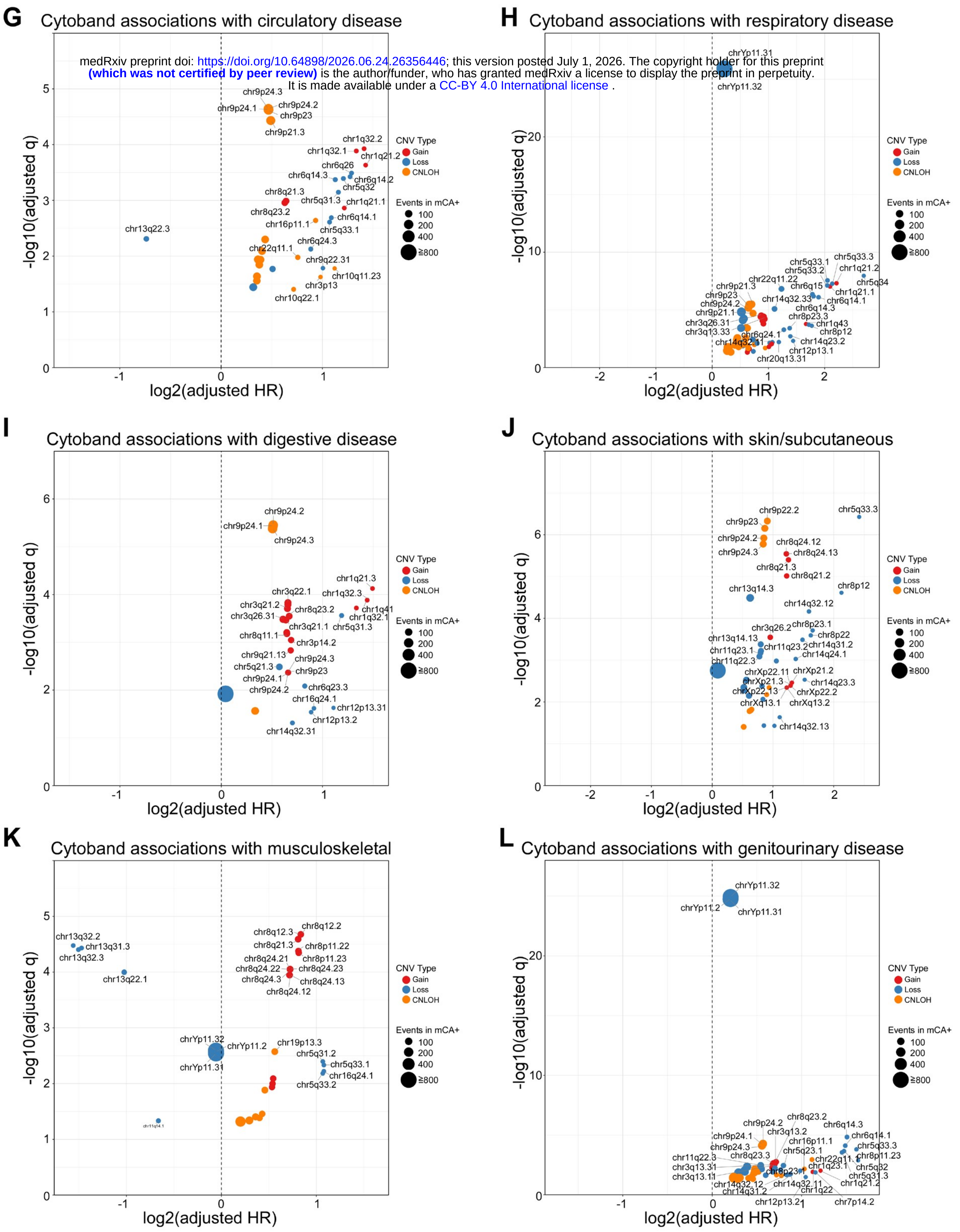

Supplementary Fig. 13

medRxiv preprint doi: https://doi.org/10.64898/2026.06.24.26356446; this version posted July 1, 2026. The copyright holder for this preprint (which was not certified by peer review) is the author/funder, who has granted medRxiv a license to display the preprint in perpetuity.
It is made available under a CC-BY 4.0 International license .

A

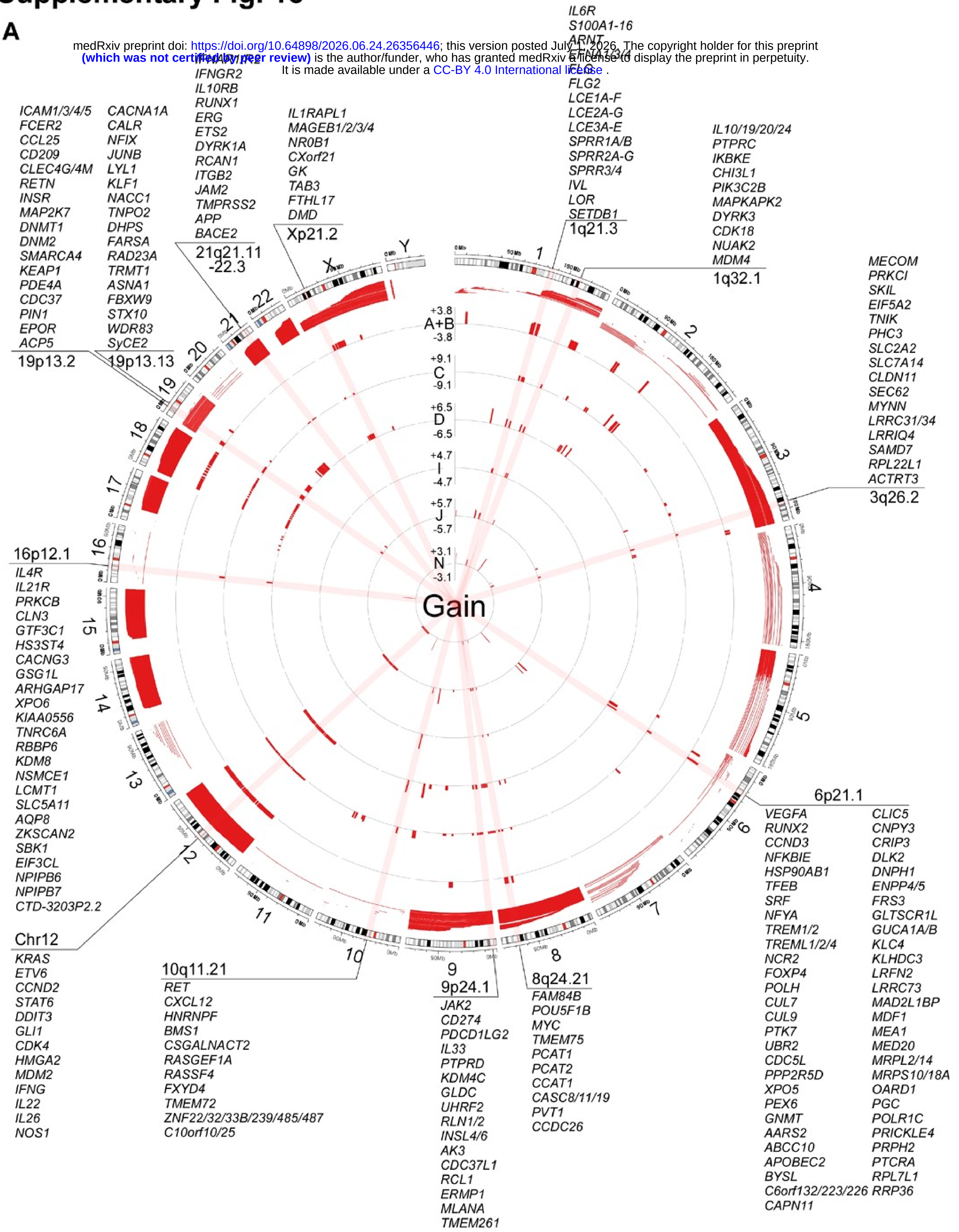

medRxiv preprint doi: https://doi.org/10.64898/2026.06.24.26356446; this version posted July 1, 2026. The copyright holder for this preprint (which was not certified by peer review) is the author/funder, who has granted medRxiv a license to display the preprint in perpetuity.
It is made available under a CC-BY 4.0 International license.

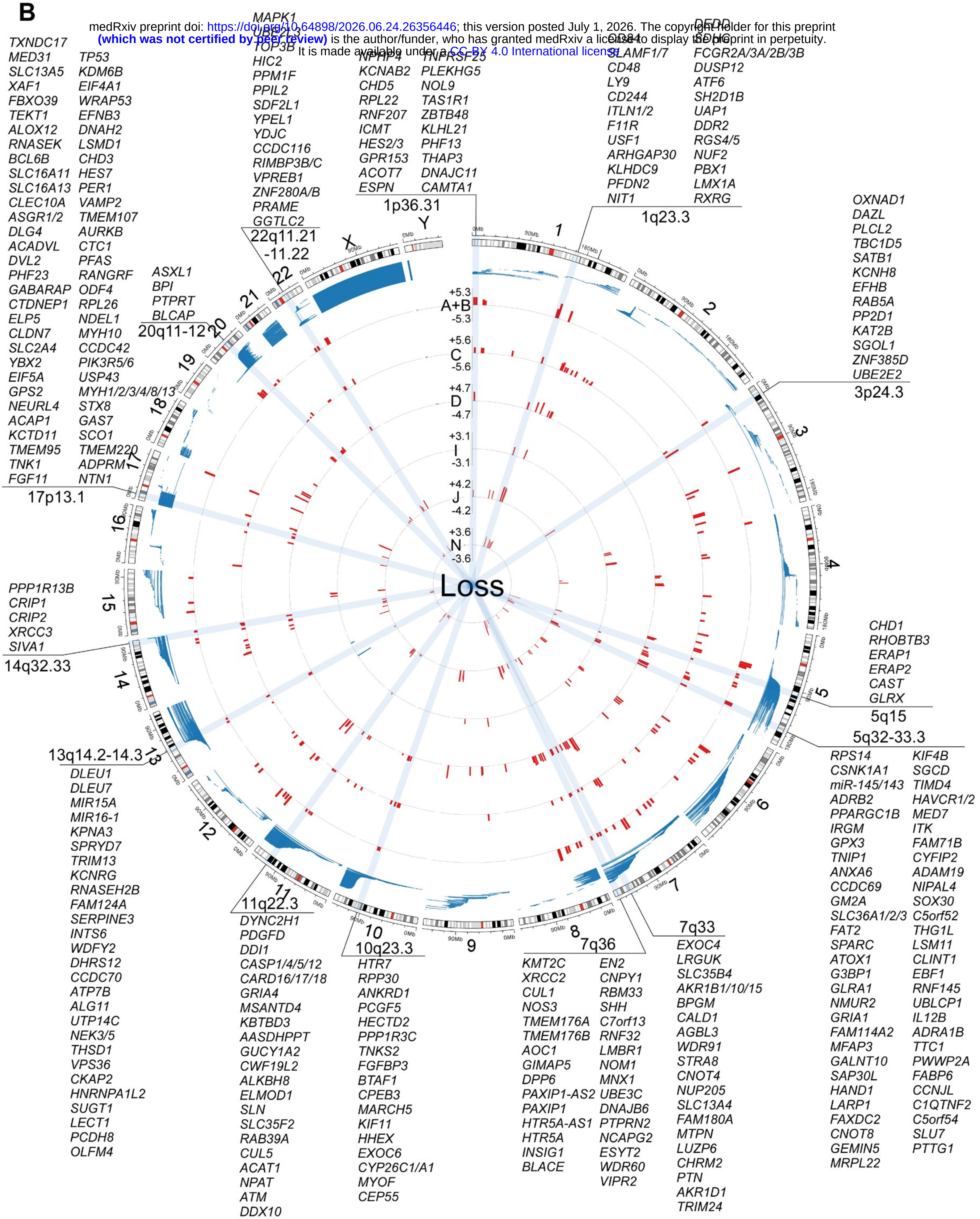

medRxiv preprint doi: https://doi.org/10.64898/2026.06.24.26356446; this version posted July 1, 2026. The copyright holder for this preprint (which was not certified by peer review) is the author/funder, who has granted medRxiv a license to display the preprint in perpetuity.
It is made available under a CC-BY 4.0 International license .

C

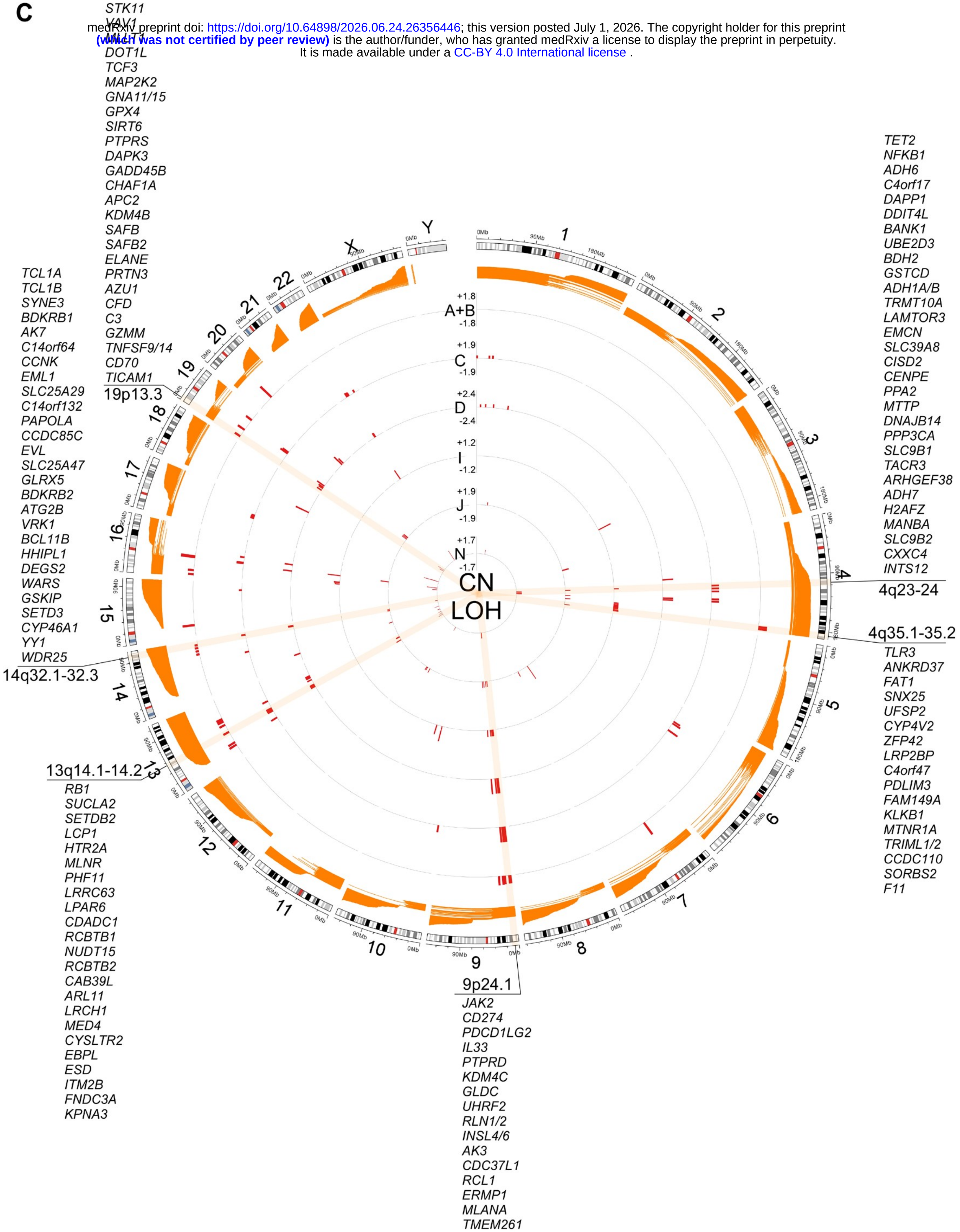

Supplementary Fig. 14

medRxiv preprint doi: https://doi.org/10.64898/2026.06.24.26356446; this version posted July 1, 2026. The copyright holder for this preprint (which was not certified by peer review) is the author/funder, who has granted medRxiv a license to display the preprint in perpetuity.
It is made available under a CC-BY 4.0 International license .

A

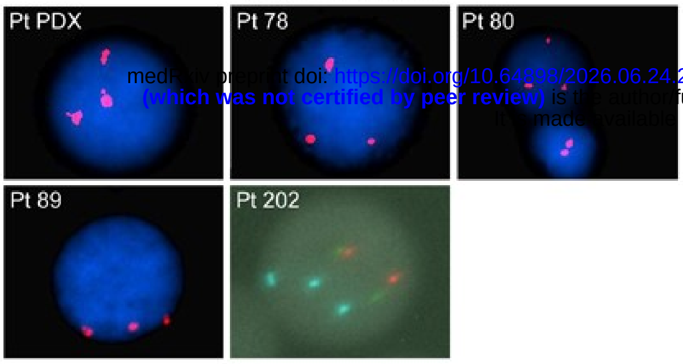

B

Genetic profiles

ID	Diagnosis	Cytogenetics	NGS
PDX	MPN	47,XY,+8[20]	*ASXL1, U2AF1*
Pt 78	MDS/MPN	47,XY,+8[19]/46,XY[1]	*NRAS, ASXL1, EZH2, RUNX1, TET2*
Pt 80	MDS	48,XX,+8,+19[20]	*ASXL1, EZH2, TET2, KRAS*
Pt 89	AML	41-48,XY,-5,del(6)(p22),+8,-17,-18,-19, add(19)(p13.1),+21,+1-4mar[cp20]	*DNMT3A, TP53, NF1*
Pt 202	AML	45,X,-Y[2]/53,X,-Yder(3)add(3)(q27)inv(3)(p24q26.2),+4,+5,der(5)t(3;5)(q26.2;q13), der(5)t(3;5)(q26.2;q14),add(6)(q15),+8, +9,-10,+13,+13,+18,der(?)t(?;6)(";q15)x2[22]	*TP53, TET2, KRAS, NCOA3*
Pt 297	AML	48,XX,t(5;6)(q33;q21),+8,+8[6]/49, idem+8[2]/46,XX[12]	*CBL, ASXL1, TET2, CDKN2A*

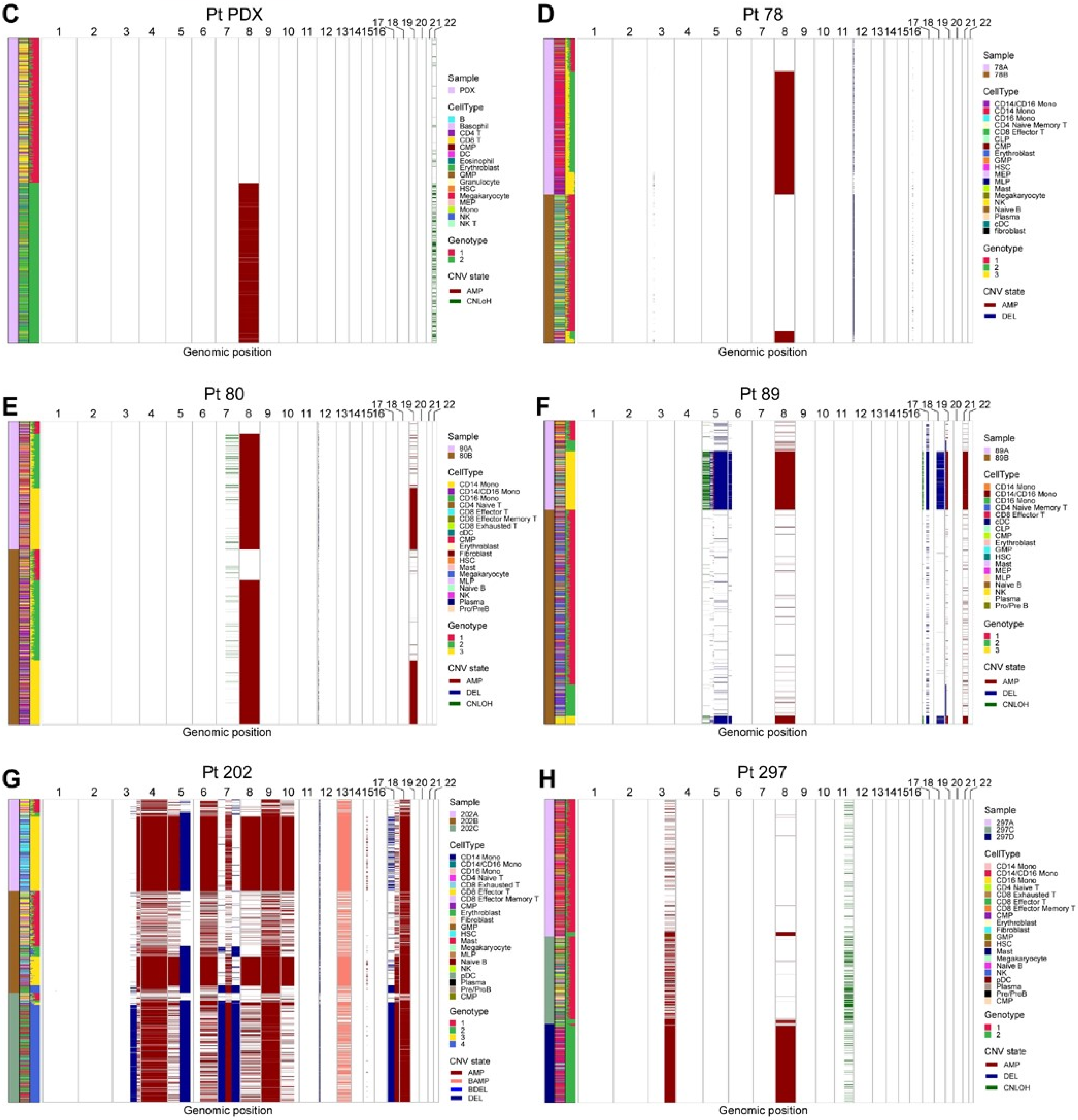

Supplementary Fig. 15

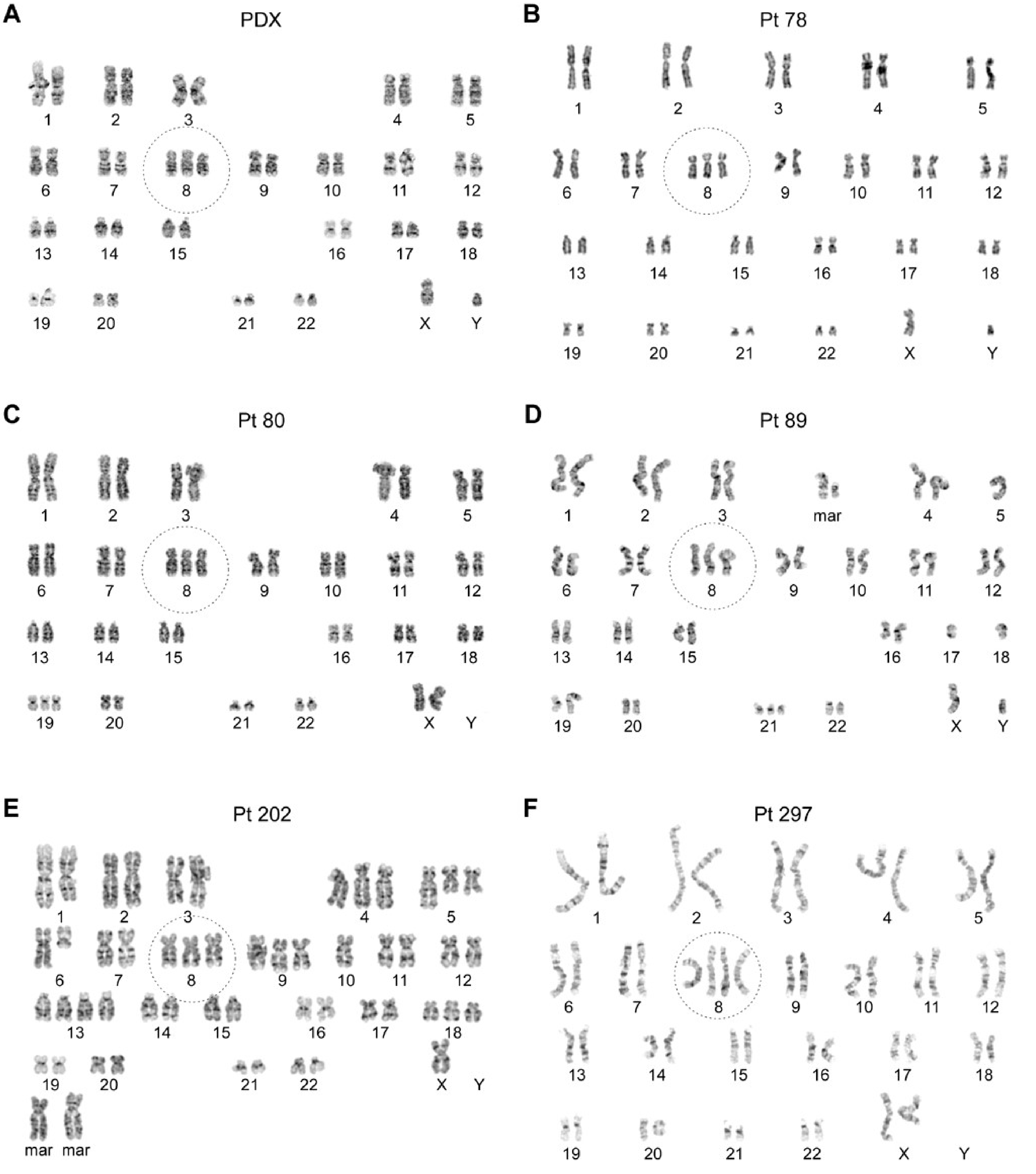

Supplementary Fig. 16

medRxiv preprint doi: https://doi.org/10.64898/2026.06.24.26356446; this version posted July 1, 2026. The copyright holder for this preprint (which was not certified by peer review) is the author/funder, who has granted medRxiv a license to display the preprint in perpetuity.
It is made available under a CC-BY 4.0 International license .

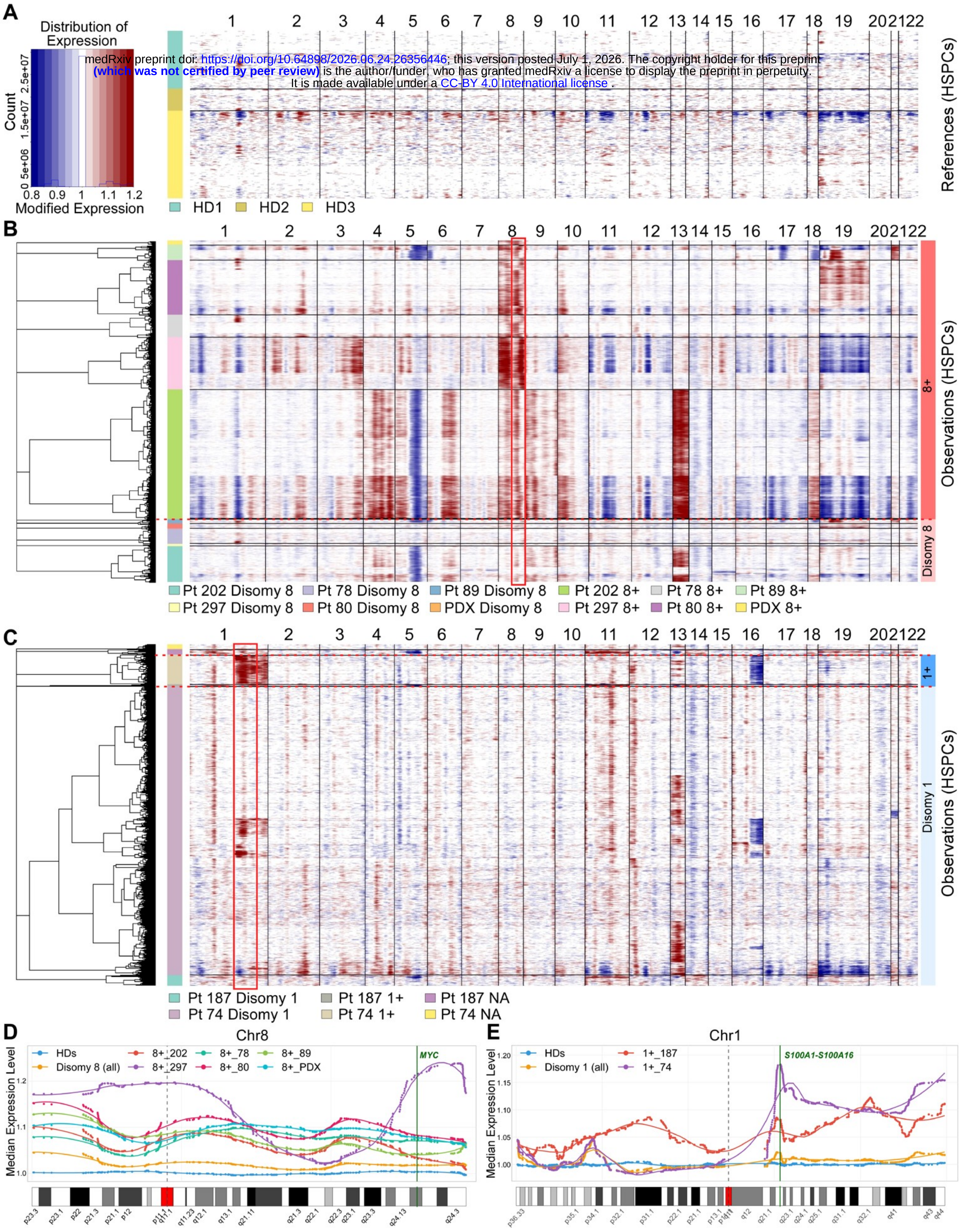

Supplementary Fig. 17

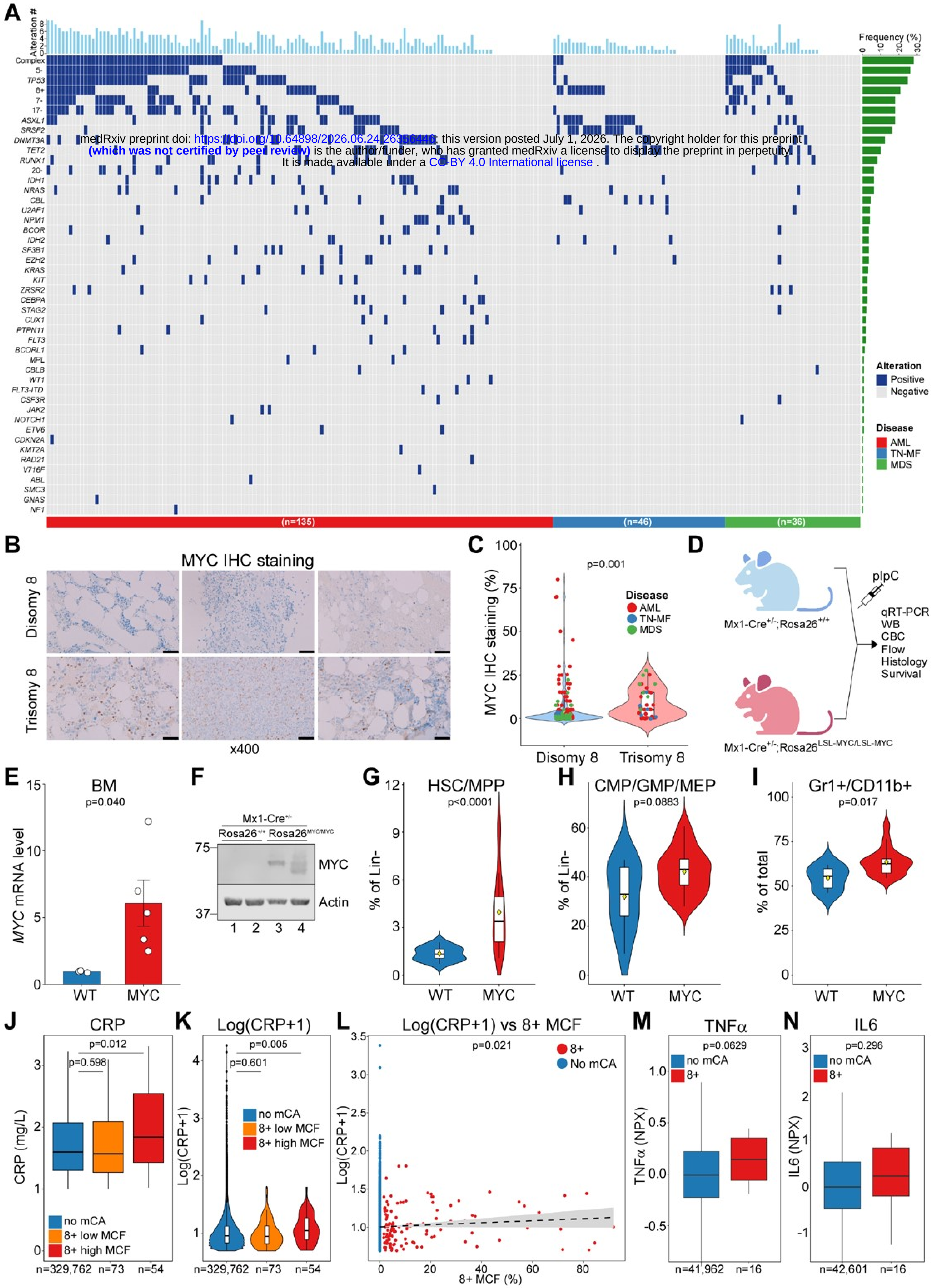

medRxiv preprint doi: https://doi.org/10.64898/2026.06.24.26356448; this version posted July 1, 2026. The copyright holder for this preprint (which was not certified by peer review) is the author/funder, who has granted medRxiv a license to display the preprint in perpetuity.
It is made available under a CC-BY 4.0 International license .

Supplementary Fig. 18

medRxiv preprint doi: https://doi.org/10.64898/2026.06.24.26356446; this version posted July 1, 2026. The copyright holder for this preprint (which was not certified by peer review) is the author/funder, who has granted medRxiv a license to display the preprint in perpetuity.
It is made available under a CC-BY 4.0 International license .

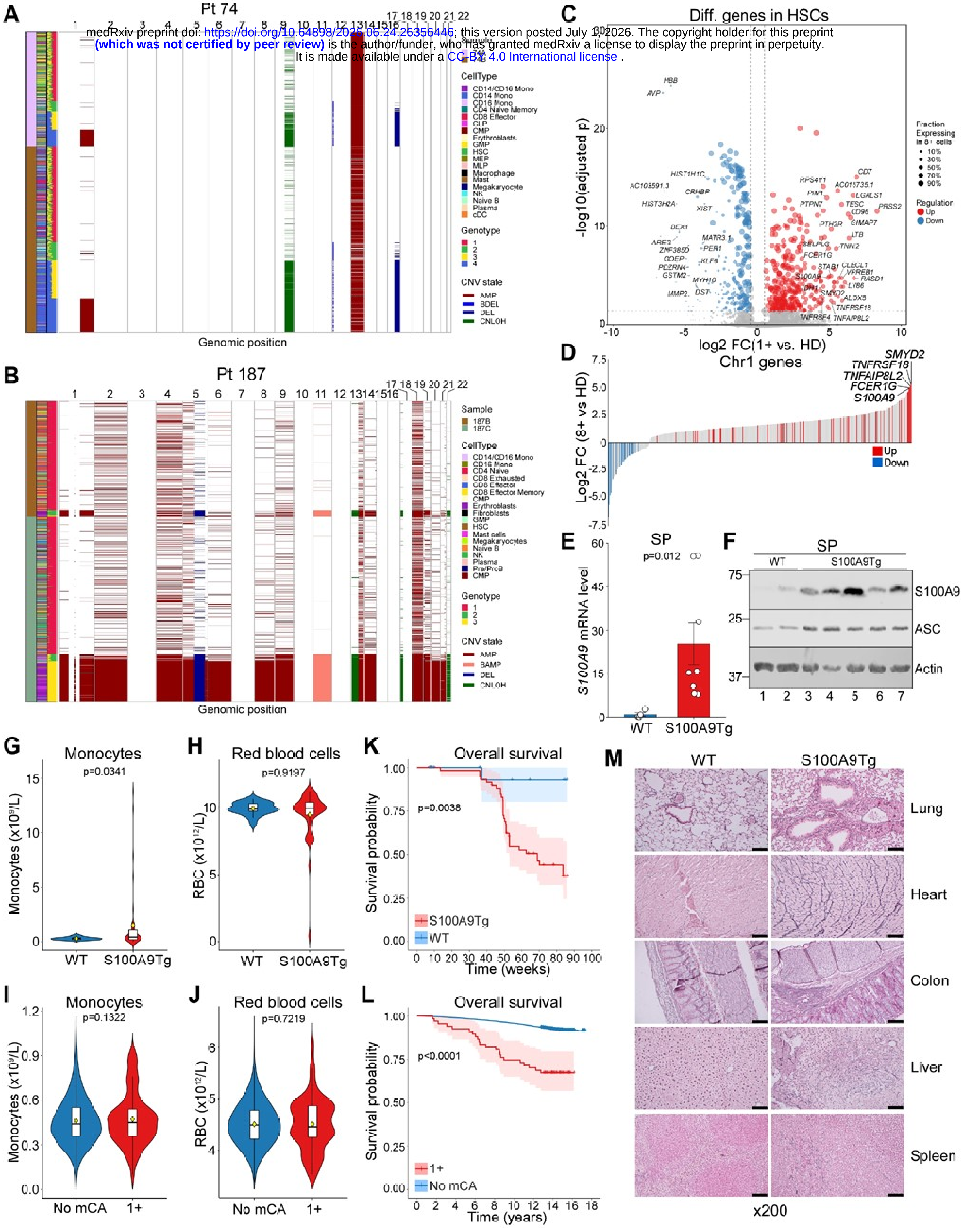

Supplementary Fig. 19

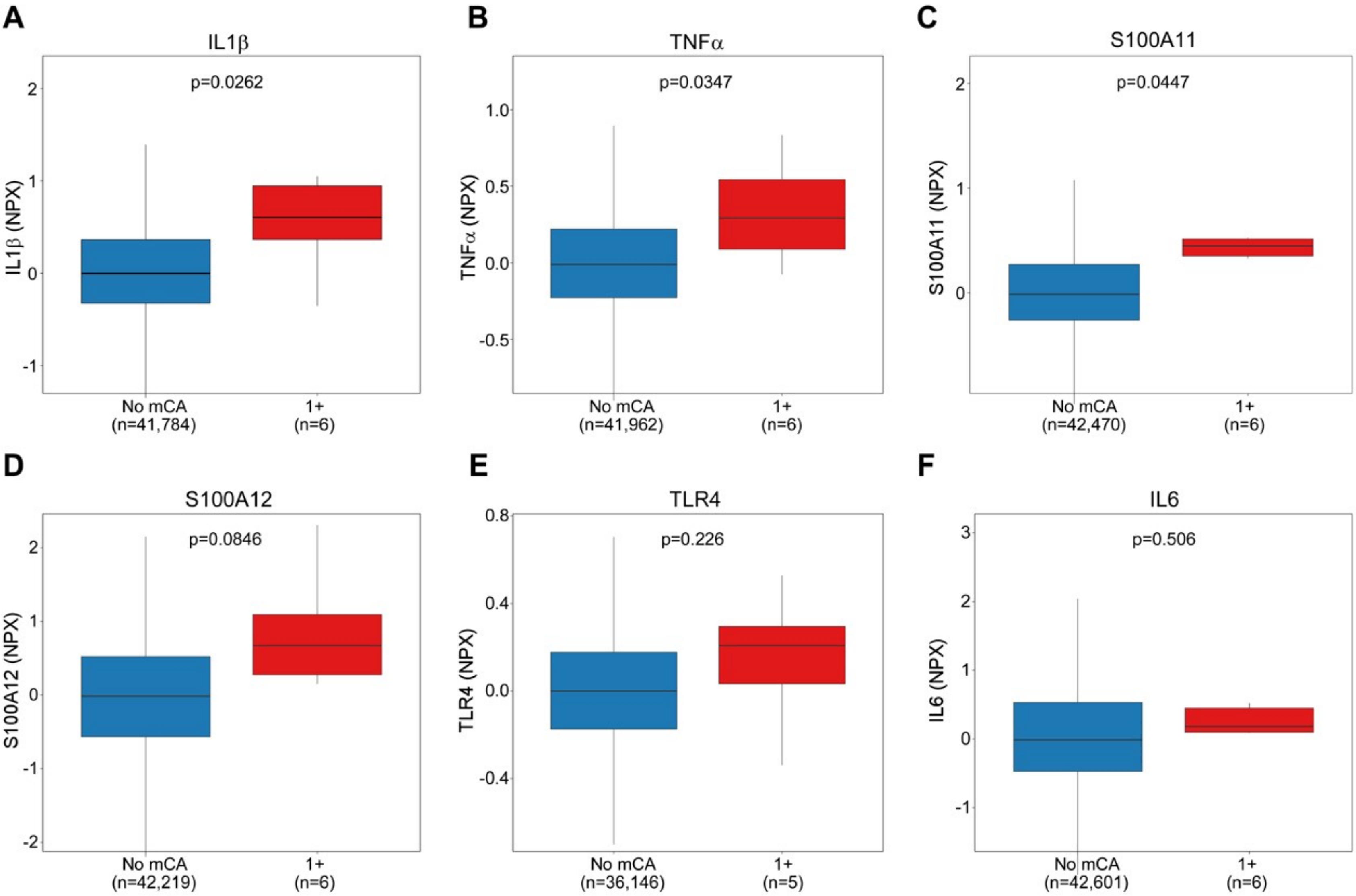

Supplement: 1 [file NIHPP2026.06.24.26356446V1-supplement-1.pdf]
